# Simultaneous real-time imaging of upper cervical cerebrospinal fluid and vascular dynamics using ultrafast inflow-weighted EPI

**DOI:** 10.1162/IMAG.a.1374

**Published:** 2026-09-22

**Authors:** Amir H. Shaker, Amir H.G. Arani, Bryn A. Martin, Aristeidis Sotiras, Cihat Eldeniz, Helia Hosseini, Joshua S. Shimony, David D. Limbrick, Jennifer M. Strahle, Arash Nazeri

**Affiliations:** Mallinckrodt Institute of Radiology, Washington University School of Medicine, St. Louis, MO, United States; Department of Neurosurgery, Washington University School of Medicine, St. Louis, MO, United States; Imaging Science Program, McKelvey School of Engineering, Washington University in St. Louis, St. Louis, MO, United States; Department of Chemical and Biological Engineering, University of Idaho, Moscow, ID, United States; Flux Neuroscience, LLC., Troy, ID, United States; Institute for Informatics, Data Science & Biostatistics, Washington University School of Medicine, St. Louis, MO, United States; Department of Radiology, Perelman School of Medicine, University of Pennsylvania, Philadelphia, PA, United States; Center for AI and Data Science for Integrated Diagnostics, Perelman School of Medicine, University of Pennsylvania, Philadelphia, PA, United States; Department of Neurosurgery, Virginia Commonwealth University, Richmond, VA, United States; Department of Biomedical Engineering, McKelvey School of Engineering, Washington University in St. Louis, St. Louis, MO, United States

**Keywords:** CSF flow, neurofluid imaging, real-time MRI, neurovascular imaging, temporal dynamics

## Abstract

Cerebrospinal fluid (CSF) circulation is tightly coupled to cerebral blood flow under the fixed-volume constraint of the cranial vault, with cardiac pulsations and respiration acting as dominant physiological drivers. Disruption of these flow dynamics has been implicated in various neurological disorders, motivating the need for imaging methods that capture CSF and vascular flow simultaneously and in real time. Conventional phase contrast MRI (PC-MRI) provides quantitative CSF velocity measurements but relies on cardiac gating and velocity encoding, which limit temporal resolution, dynamic range, and sensitivity to non-cardiac fluctuations. Here, we implement a single-slice ultrafast inflow-weighted echo-planar imaging (EPI), in which signal intensity reflects the replacement of saturated spins by freshly inflowing, unsaturated water molecules, yielding higher signal with higher flow velocity. This allows simultaneous assessment of arterial inflow, venous outflow, and CSF motion at high temporal resolution. Ultrafast EPI was acquired at the C2–C3 spinal level, enabling real-time imaging of CSF flow dynamics in the cervical spinal canal alongside arterial and venous blood flow in major cervical vessels (frame rate: 21.7 Hz). Furthermore, intrinsic arterial signal fluctuations were leveraged as a timing reference to reconstruct cardiac-resolved CSF dynamics for individual cardiac cycles without external physiological recordings. Frequency-domain analysis revealed distinct spectral signatures in CSF flow compared with neurovascular flow, including broadened cardiac peaks and enhanced respiratory modulation, particularly within the ventral spinal canal. In contrast, dorsal CSF showed increased power within the cardiac frequency band, higher coherence with cervical vasculature at the cardiac frequency, and reduced non-cardiac contributions. Time-domain analysis showed strong correlation between CSF flow waveforms derived from ultrafast EPI and PC-MRI. Beyond ensemble-averaged waveforms, ultrafast EPI enabled beat-to-beat analysis, revealing substantial cycle-to-cycle variability in CSF flow that is not captured by time-averaged gated approaches. Together, these findings establish ultrafast EPI as a rapid, complementary framework to PC-MRI, enabling real-time neurofluid imaging with integrated time- and frequency-domain characterization of CSF and neurovascular flow dynamics.

## 1 Introduction

Cerebrospinal fluid (CSF) circulation plays a critical role in maintaining brain fluid homeostasis and clearing of metabolic waste (Kelley & Thomas, 2023; Rasmussen et al., 2022). Disruptions in this tightly regulated system have been implicated in various brain disorders ranging from hydrocephalus, idiopathic intracranial hypertension to stroke and neurodegenerative disorders (Bothwell et al., 2019; Menéndez González, 2023; Rasmussen et al., 2022). The rigid cranial vault imposes a fixed-volume constraint (Monro–Kellie doctrine), such that CSF flow dynamics are inseparably linked to cerebral blood flow (Nazeri et al., 2025; Wang et al., 2022). Within this confined system, cardiac pulsations and respiratory cycles are the primary drivers of CSF and cerebral blood flow (Dreha-Kulaczewski et al., 2015, 2017). Accordingly, capturing these interactions in real time through simultaneous imaging of vascular and CSF flow is critical for understanding neurofluid physiology in health and its pathological disruption in disease.

Cardiac-gated phase-contrast (PC) MRI has long served as the standard imaging approach to quantify CSF velocity and flow across the cardiac cycle (Wymer et al., 2020; Yamada et al., 2015). However, conventional PC-MRI relies on cardiac gating, which resolves CSF flow across the cardiac cycle as an average of multiple heartbeats, thereby overlooking the real-time fluctuations driven by respiration and other non-cardiac factors (Chen et al., 2015; Spijkerman et al., 2019). Real-time PC-MRI, by contrast, circumvents the need for gating and allows dynamic flow assessment (Chen et al., 2015; Liu et al., 2024; Yildiz et al., 2017), enabling evaluation of respiratory and other non-cardiac drivers of CSF flow, as well as characterization of flow variability across cardiac cycles. Nonetheless, the dynamic range of both conventional and real-time PC methods is constrained by velocity encoding (VENC) setting, which sets the maximum measurable velocity without aliasing (Wymer et al., 2020). Lowering the VENC thresholds can enhance phase signal-to-noise ratio (SNR) for detecting slow flow, but it also increases the risk of velocity aliasing (Nayak et al., 2015). While recent studies demonstrate the feasibility of simultaneous blood and CSF flow assessment (Liu et al., 2025; Rivera-Rivera et al., 2025), the velocity constraints imposed by fixed VENC selection require careful trade-offs between arterial, venous, and CSF flow sensitivity in PC-MRI.

To overcome these limitations, alternative imaging strategies are being explored that allow simple CSF flow imaging using fast imaging methods (Dreha-Kulaczewski et al., 2015; Fultz et al., 2019). Inflow-weighted approaches exploit flow-related signal enhancement. Freshly entering, unexcited CSF produces high signal intensity, whereas stationary fluid signal within the slab remains suppressed because its long T1 prevents appreciable recovery of longitudinal magnetization at short repetition times (TRs) (Diorio et al., 2024; Dreha-Kulaczewski et al., 2015). Previous studies employing whole-brain echo-planar imaging (EPI) have successfully mapped unidirectional CSF flow in a few edge slices, restricted to flow entering the imaging slab. However, these methods require extended acquisition times and offer relatively low temporal resolution (250–500 ms) (Ashenagar et al., 2025; Diorio et al., 2024; Fultz et al., 2019; Nair et al., 2024; Williams et al., 2023). Other studies using ultrashort TRs (<50 ms) have demonstrated that ultrafast EPI can capture vascular flow dynamics (Bianciardi et al., 2016; Whittaker et al., 2022).

**Fig. 1.**
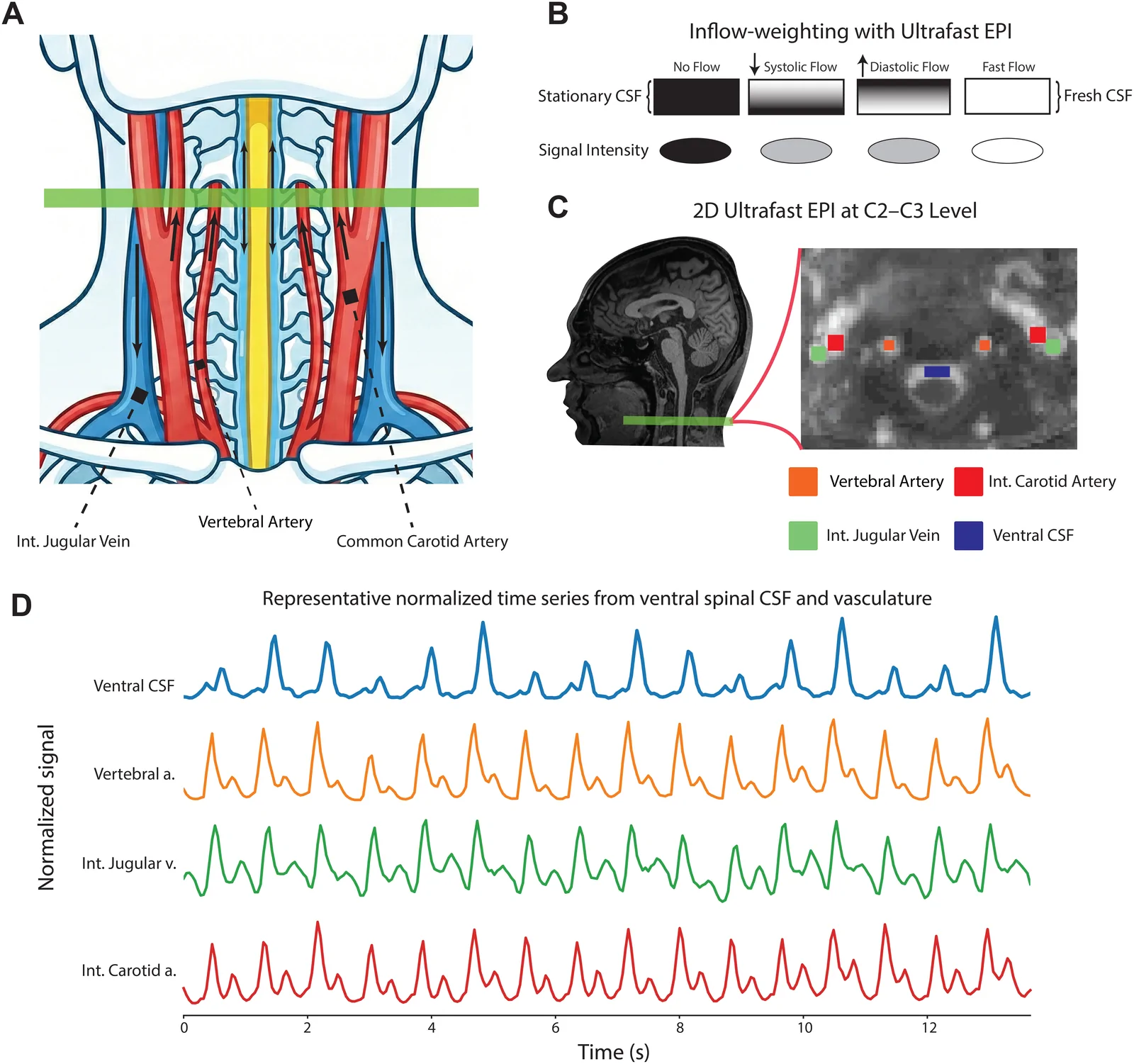
MR physics basis of ultrafast EPI and its application for capturing cervical vascular and CSF pulsatility. (A) Coronal schematic of cervical neurovascular anatomy at the C2–C3 level, showing the spatial relationship between vertebral arteries, carotid arteries, internal jugular veins, and the spinal canal. The green bar denotes the 2D ultrafast EPI slice position. (B) MR physics principle underlying ultrafast EPI inflow weighting. Because the sequence uses a very short TR and large flip angle with repeated excitations, stationary water molecules remain saturated and yield near-zero signal. In contrast, through-plane flow brings fresh, unsaturated spins into the imaging slice, producing bright signal. (C) Example 2D ultrafast EPI scan at C2–C3 showing the temporal standard deviation of the ultrafast EPI signal over time (log scale). Regions of interest include the vertebral arteries (orange), internal carotid arteries (red), internal jugular veins (green), and ventral spinal canal CSF (blue). (D) Representative normalized time series from ventral CSF and vascular regions of interest acquired with ultrafast EPI. Signals were normalized for visualization of temporal features, as arterial pulsation amplitudes substantially exceed those of CSF.

Building on these approaches, we implement a single-slice ultrafast inflow-weighted EPI that enables self-gated CSF flow imaging at a high sampling rate (temporal resolution: 46 ms) with an in-plane spatial resolution of 1.3 × 1.3 mm^2^ (see Fig. 1 for an overview of the approach). This high spatiotemporal resolution is achieved by single slice imaging and narrowing the field of view using saturation bands. In addition, the wide dynamic range of ultrafast EPI enables simultaneous sensitivity to both neurovascular blood flow and CSF motion. This facilitates interrogation of interactions between venous outflow (internal jugular veins), arterial inflow (internal carotid and vertebral arteries), and CSF dynamics across both time and frequency domains. In particular, arterial signal fluctuations can be used to extract the cerebral arterial input function, allowing reconstruction of CSF cardiac phases without reliance on external gating. As with other inflow-weighted imaging techniques, ultrafast single-slice EPI captures CSF flow dynamics as temporal changes in voxel signal intensity (Fultz et al., 2019). The relationship between inflow-weighted signal and flow velocity is complex and potentially nonlinear (Ashenagar et al., 2025; Diorio et al., 2024), limiting direct velocity quantification. Nevertheless, we show that the CSF flow waveform derived from ultrafast EPI approximates that of cardiac-gated PC-MRI, suggesting the potential for calibrating signal intensity to velocity. Thus, our approach complements conventional PC-MRI by enabling ultrafast CSF flow imaging independent of velocity encoding, reducing sensitivity to dynamic range limitations and phase noise, while permitting simultaneous assessment of neurovascular and CSF flow dynamics.

## 2 Methods and Materials

### 2.1 Participants

Adult participants were recruited from the Washington University in St. Louis community. Exclusion criteria were primary CSF disorders (e.g., idiopathic intracranial hypertension, hydrocephalus, CSF leak), neurodegenerative disorders, major systemic illnesses, uncontrolled diabetes or hypertension, or any contraindication for MRI. All experimental procedures were approved by the Washington University School of Medicine Institutional Review Board. Written informed consent was obtained from all participants, and all participants received financial compensation for participation.

### 2.2 MRI acquisition

All images were acquired using a 3T Siemens Prisma scanner (software version: XA30, Siemens Healthineers, Erlangen, Germany) equipped with a 64-channel head coil. In a subset of participants (n = 16), cardiac (photoplethysmography) and respiratory (respiratory belt) signals were continuously acquired at 40 Hz using an MR-compatible physiological monitoring system integrated with the scanner. All relevant code developed for this study is publicly available (https://github.com/BRAFTI/surf-epi).

#### 2.2.1 Ultrafast EPI

Ultrafast single-slice EPI images were acquired with large flip angle and short TR to enhance inflow weighting (Diorio et al., 2024) and achieve high temporal resolution (Fig. 1). Two coronal saturation bands (8.4 cm thickness) were applied to enable small field-of-view imaging without aliasing. The anterior band was positioned over the anterior nasopharynx and neck/face soft tissues, and the posterior band over the posterior cervical soft tissues. Ultrafast EPI acquisitions were performed at the C2–C3 vertebral disk level (Fig. 1), oriented perpendicular to the spinal cord under task-free conditions, allowing free breathing and the natural occurrence of physiological fluctuations without external stimuli or controlled breathing instructions. Acquisition parameters were as follows: TR: 46 ms (sampling rate: 21.7 Hz), TE: 14 ms, flip angle: 90°, slice thickness: 5 mm, in-plane resolution: 1.3 × 1.3 mm^2^, FOV: 125 × 94 mm^2^, and 3,000 time points (141 seconds). Parallel imaging was employed using modified sensitivity encoding (mSENSE) (Pruessmann et al., 1999) with an acceleration factor of 2 and phase-encoding partial Fourier of 6/8. In a subset of participants (n = 16), an additional ultrafast EPI acquisition with identical parameters was obtained at the craniocervical junction, immediately below the foramen magnum. To assess the effect of temporal resolution on signal quality, additional ultrafast EPI acquisitions were obtained in five participants at TRs of 33, 70, and 100 ms. The 70- and 100-ms acquisitions used the same sequence geometry, spatial resolution, and anterior/posterior saturation-band configuration as the standard 46 ms acquisition, with only the TR adjusted. To achieve TR = 33 ms (30-Hz frame rate), the sequence was modified by reducing the matrix size/in-plane resolution to 2 × 2 mm^2^ and using only the anterior saturation band.

#### 2.2.2 Phase-contrast MRI

PC-MRI was performed at the C2–C3 level using the same slice orientation as ultrafast EPI, with a velocity encoding (VENC) of 8 cm/s. Acquisition parameters were as follows: TR: 66.6 ms, TE = 7.21 ms, flip angle = 10°, slice thickness: 5 mm, in-plane resolution: 0.6 × 0.6 mm^2^, FOV: 160 × 160 mm^2^, calculated phases = 40, two signal averages, acquisition time: 2:52.

### 2.3 Ultrafast EPI analysis

For all ultrafast EPI analyses, regions of interest (ROIs) were defined using FSLeyes (part of FSL v6.0.7) (Jenkinson et al., 2012), including the ventral spinal canal, dorsal spinal canal (when present), vertebral arteries, internal carotid arteries, and internal jugular veins. CSF ROIs were defined using temporal standard deviation maps generated from the entire ultrafast EPI time series, which highlighted dynamically varying CSF and vascular signals relative to the stationary spinal cord and osseous structures. The ventral CSF ROI was placed immediately anterior to the spinal cord, with approximately one voxel excluded from each lateral edge to avoid inclusion of the internal vertebral venous plexus. The dorsal CSF ROI encompassed the clearly visible dorsal and dorsolateral subarachnoid space when present. To assess intra-rater reliability, one rater (A.H.S.) repeated the ventral and dorsal CSF ROI delineations using the same standardized instructions, and Dice similarity coefficients were calculated between the original and repeated ROIs. All signal processing described below was performed using SciPy (v1.11.0).

#### 2.3.1 Power spectral analysis

Power spectral density (PSD) was computed using the welch function from the *scipy.signal* library, applied to the mean time series from each ROI. To account for inter-individual variability in respiratory and heart rates, power spectra were rescaled such that the respiratory and cardiac peaks were mapped to fixed normalized frequencies of 0.25 and 1, respectively. This normalization enabled comparison of physiological signals across participants independent of absolute rate differences in respiratory and heart rates (Supplementary Fig. S1). For each subject, respiratory rate was estimated from the ventral CSF ROI power spectrum, whereas heart rate was estimated from the average power spectrum across all CSF and vascular ROIs. The frequency axis was then linearly rescaled to align these peaks to fixed reference values (0.25 and 1.0, respectively) using

$$f_{norm}\;=a\;f\;+\;b,$$

where the coefficients were determined by enforcing f_norm_ (respiratory rate) = 0.25 and f_norm_ (heart rate) = 1.0. Each spectrum was interpolated using *scipy.interpolate.interp1d* for resampling onto a shared frequency grid. To assess physiological coupling between CSF and vascular signals, magnitude-squared coherence was calculated using the *coherence* function from *scipy.signal*, with nperseg = 500, applied to pairs of region-averaged time series. Coherence spectra were rescaled using the same subject-specific frequency alignment and interpolated to a common frequency grid for group-level averaging and analysis.

#### 2.3.2 Cardiac cycle detection and reconstruction

Voxel-wise arterial waveforms (ultrafast EPI signal time series) were extracted from vertebral artery ROIs. These waveforms were standardized by z-scoring. Cardiac systolic peaks were identified using the *find_peaks* function from the *scipy.signal* module with a minimum peak height of 0.5 (arbitrary units) and a minimum inter-peak interval of 670 ms to suppress false detections. The voxel with the greatest number of systolic peaks was selected for further analysis; in cases of ties, preference was given to the voxel with the highest power in the 0.8–1.2 Hz frequency (cardiac) band, ensuring selection of the voxel with the strongest cardiac frequency-driven signal. Cardiac cycles were defined as the interval spanning seven time points (~330 ms) before one vertebral artery systolic peak to seven time points before the next, with the preceding window assumed to capture the CSF diastolic phase. For each voxel, time series segments of average cycle length were extracted, aligned to systolic peaks, and averaged to yield a representative gated waveform.

### 2.4 Cardiac cycle-resolved CSF flow metrics

Within the ventral and dorsal spinal canal CSF ROIs, the following CSF flow metrics were calculated: (i) CSF *systole/diastole flow ratio (unitless)*, defined as the ratio of the peak systolic signal intensity to the mean signal intensity during late diastole, computed over the final four diastolic time points of the cardiac cycle within the CSF ROI; (ii) *beat-to-beat variability measures* (unitless), computed as the coefficients of variation across cardiac cycles for (a) peak systolic signal intensity, (b) late diastolic signal intensity, and (c) the systole/diastole signal intensity ratio; (iii) *the fraction of extreme cycles*, defined as the proportion in which peak systolic or late-diastolic signal exceeded twice the corresponding amplitude of the ROI-averaged waveform. To account for ROI-dependent measurement variability, we quantified signal quality metrics for each CSF ROI on a voxel-wise basis and then averaged across voxels within each ROI. Temporal SNR (tSNR) was defined as the ratio of the temporal standard deviation in each CSF voxel to that of a background reference (vertebral body). Temporal contrast-to-noise ratio (tCNR) was defined as the peak-to-trough signal variation across the reconstructed cardiac cycle in each voxel, normalized by the background temporal standard deviation. ROI size (voxel count) was also included to account for potential partial volume effects.

### 2.5 Comparison of CSF flow waveforms derived from ultrafast EPI and PC-MRI

CSF flow waveforms reconstructed from ultrafast EPI data were compared with absolute waveforms extracted from PC-MRI to assess agreement independent of flow direction. In both modalities, mean signal waveforms were extracted from the ventral spinal canal ROI. The ultrafast EPI CSF flow waveform was interpolated to match the temporal resolution of the PC-MRI data. The waveforms were aligned by temporally shifting the PC-MRI signal, with the PC-MRI systolic peak constrained to occur after the arterial peak in the ultrafast EPI signal and within 35% of that participant’s mean cardiac cycle duration. For visualization purposes, both waveforms were linearly rescaled to a common amplitude range. Similarity between CSF flow waveforms derived from the two modalities was quantified using the Pearson correlation coefficient.

### 2.6 Vascular inflow-signal modeling

To characterize the dependence of vascular inflow-weighted signal on velocity and repetition time (TR), we modeled laminar flow through a vessel oriented perpendicular to a slice of thickness (Δz = 5) mm, following previously described approaches (Diorio et al., 2024; Gao et al., 1988). A fully developed Poiseuille profile was assumed:

$$v\left(r\right)=v_{\text{peak}}\left(1-\frac{r^{2}}{R^{2}}\right),$$

where $v_{\text{peak}}$ is the centerline velocity and (R) is the vessel radius. Under this profile, equal velocity intervals occupy equal fractions of the vessel cross-sectional area; therefore, velocities are uniformly distributed between 0 and $v_{\text{peak}}$ . For a 90° flip angle and short TR, the normalized signal was approximated as the fraction of spins replaced between successive excitations. Relaxation during TR was neglected, and complete spin replacement was assigned a normalized signal of 1. The velocity required for complete replacement of spins within one TR was

$$v_{\text{max}}=\frac{\Delta \;z}{\text{TR}}.$$

The velocity-dependent signal was, therefore, defined as

$$S\left(v\right)=\min\left(1,\frac{v}{v_{\text{max}}}\right).$$

Averaging the signal over the vessel cross-section yielded

$$\begin{array}{l} \bar{S}=\frac{1}{v_{\text{peak}}}\int_{0}^{v_{\text{peak}}}\min\;\text{​}\left(1,\;\frac{v}{v_{\text{max}}}\right)dv\\ \;\;\;\;\;=\{\begin{array}{cc} \frac{v_{\text{peak}}}{2\;v_{\text{max}}} & v_{\text{peak}}\leq v_{\text{max}}\\ 1-\frac{v_{\text{max}}}{2\;v_{\text{peak}}} & v_{\text{peak}}>v_{\text{max}} \end{array}. \end{array}$$

The model was evaluated for TRs of 33, 46, 70, and 100 ms and peak velocities ranging from 0 to 100 cm/s. Signal values were normalized to the signal corresponding to complete spin replacement.

### 2.7 Statistical analysis

All statistical analyses were performed in R (v4.3.1). To validate our self-gated cardiac cycle detection, we compared interbeat intervals derived from vertebral artery peak systole time points in ultrafast EPI with those obtained from pulse oximeter recordings (index finger) in the physiological log. Agreement between the two methods was assessed using Bland–Altman analysis. The intraclass correlation coefficient (ICC) was additionally calculated to quantify the consistency of the measurements. Paired t-tests were used for point-wise comparisons of ventral versus dorsal CSF flow dynamics, as well as for comparisons between ventral CSF and neurovascular signals in the power spectral density domain across the frequency spectrum (0.02–5 Hz). For all spectral analyses, *p* values were corrected for multiple comparisons across frequencies using the false discovery rate, with *p*_FDR_ < 0.05 considered statistically significant. Extracted CSF waveform and variability metrics were compared between ventral and dorsal compartments using paired t-tests. To assess potential confounding by ROI-related measurement factors, we repeated these comparisons in separate linear models adjusting for tSNR, tCNR, or ROI size.

#### 2.7.1 Reliability analysis

To evaluate within-scan reproducibility, each acquisition was split into first and second halves and the full cardiac-cycle detection and metric extraction pipeline were applied independently to each. Split-half reliability was quantified using ICC(2,1) (two-way random, absolute agreement).

#### 2.7.2 Multi-TR analysis

To ensure equal acquisition duration across TR conditions, each time series was truncated to 84 seconds. Ventral CSF tSNR was calculated as the ratio of the temporal SD of the CSF signal to that of the background vertebral body signal. tCNR was defined as the peak-to-trough signal variation across the reconstructed cardiac cycle within each CSF ROI, normalized by the temporal SD of the background vertebral body signal. tCNR was also calculated for the internal jugular veins, vertebral arteries, and internal carotid arteries. Differences between TR conditions were assessed using pair-wise paired t-tests. Monotonic dependence on TR was evaluated by fitting, for each subject, an ordinary least-squares regression of each metric against TR (33, 46, 70, and 100 ms) and testing the resulting slopes against zero using a one-sample t-test. This analysis included subjects with at least three of the four TR acquisitions (n = 5); one subject was missing the 70-ms acquisition. All *p* values were nominal and unadjusted for multiple comparisons.

## 3 Results

### 3.1 Imaging cohort characteristics and analysis pipeline verification

Twenty-seven adults underwent ultrafast EPI imaging at C2–C3. One participant was excluded due to motion artifact, leaving 26 participants for analysis (mean age, 28.4 ± 5.8 years; range, 21–44 years; 12 female). Dorsal CSF was visible in 22 participants, who were included in the ventral–dorsal CSF comparisons. Sixteen participants also underwent an additional ultrafast EPI acquisition at the craniocervical junction (CCJ). Five participants underwent ultrafast EPI acquisitions at multiple repetition times for the multi-TR analysis.

**Fig. 2.**
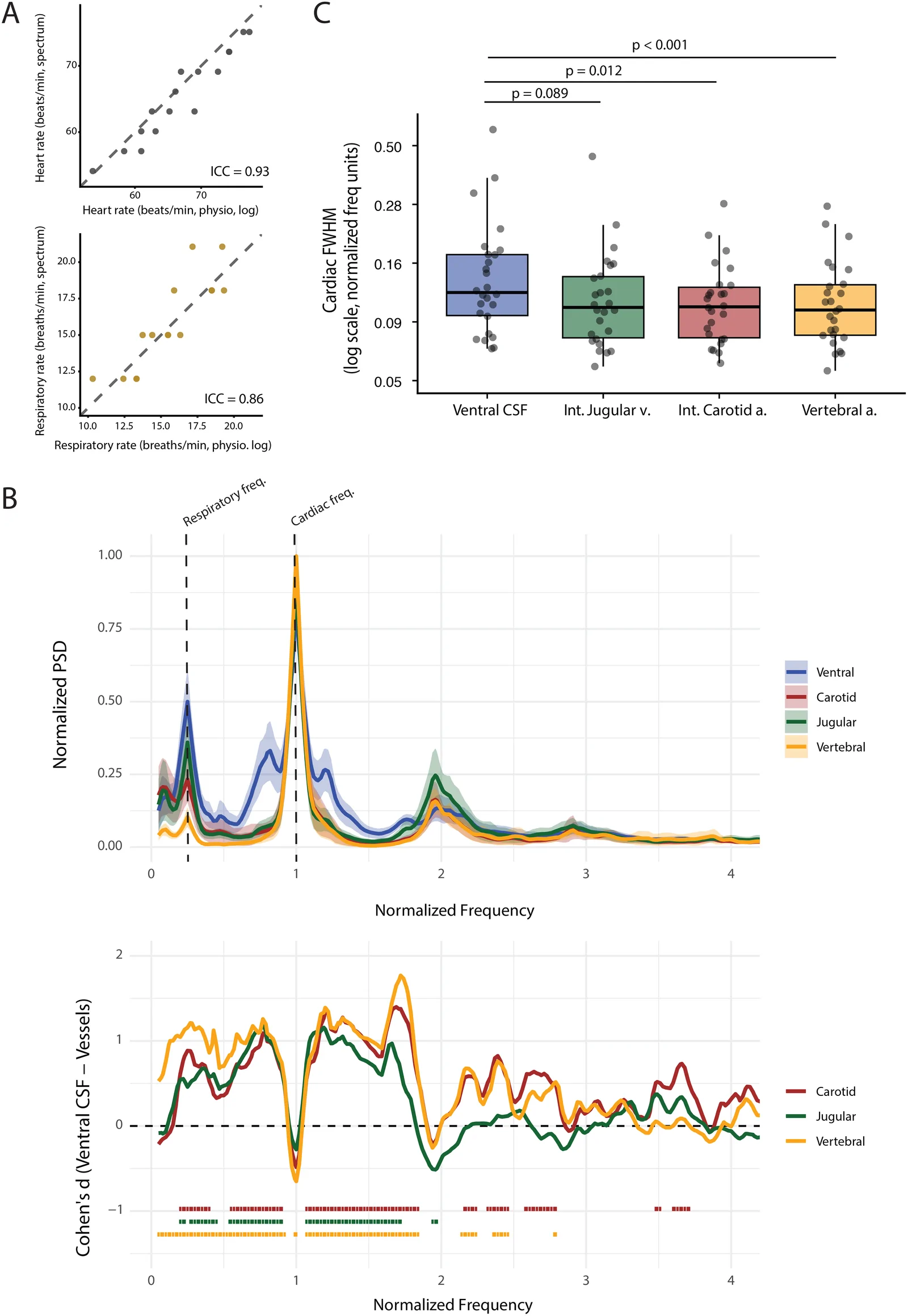
Spectral signatures reveal distinct flow dynamics in CSF and neurovasculature at **C2–C3**. (A) Agreement between physiological monitoring and ultrafast EPI-derived estimates of heart rate (HR, top) and respiratory rate (RR, bottom), demonstrating high concordance across participants. Solid lines indicate linear fits; shaded regions denote 95% confidence intervals. (B) *Top*, group-averaged normalized power spectral density (PSD) of ultrafast EPI signals from ventral CSF, the internal jugular vein, internal carotid artery, and vertebral artery. To account for interindividual variation in respiratory and heart rates, frequencies were rescaled such that the cardiac fundamental frequency equaled 1 and the respiratory frequency was set to 0.25. Shaded bands indicate 95% confidence intervals. *Bottom*, frequency-resolved Cohen’s *d* effect sizes for ventral CSF relative to each vascular compartment. Positive values indicate greater power in ventral CSF, and colored markers indicate frequencies surviving false-discovery-rate (FDR) correction. (C) Cardiac spectral broadening, quantified as the full width at half maximum (FWHM) of the cardiac peak, across ventral spinal canal CSF and major cervical vessels (y-axis is log-scale). CSF exhibits broader cardiac spectral peaks compared with arterial/venous flow signals, consistent with greater temporal dispersion. (C) Group-averaged normalized power spectral density (PSD) of ultrafast EPI signals from ventral CSF, internal jugular vein, internal carotid artery, and vertebral artery (top), demonstrating distinct frequency content across fluid compartments.

CSF ROI delineation showed high intra-rater reproducibility. Dice similarity coefficients between the initial and repeat delineations were 0.91 ± 0.096 for ventral CSF and 0.84 ± 0.11 for dorsal CSF, indicating excellent and good reproducibility, respectively. Average cardiac and respiratory rates could be accurately estimated directly from the power spectra without the need for physiological log data (intraclass correlation coefficients: ICC_Card_ = 0.93; ICC_Resp_ = 0.86; Fig. 2A).

### 3.2 Spectral signatures reveal distinct flow dynamics in CSF and neurovasculature

For frequency domain analysis, we first examined the power spectra of CSF flow signal within the ventral spinal canal and blood flow signals from the internal jugular vein, vertebral artery, and internal carotid artery at the C2–C3 level. The averaged spectrum revealed prominent peaks at respiratory and cardiac frequencies, along with higher-order cardiac harmonics, in both ventral spinal canal CSF and neurovascular ROIs including the internal jugular veins, vertebral arteries, and internal carotid arteries (Fig. 2B). Unlike the vascular flow spectra, the ventral CSF exhibited distinctive side peaks flanking the fundamental cardiac frequency. Point-wise comparisons of the power spectrum showed significantly greater power in the ventral CSF at the flanks of the fundamental cardiac frequency compared with all vascular ROIs (*p*_FDR_ < 0.001), suggesting it reflects neurophysiological fluid dynamics unique to the CSF rather than heart rate variability. To determine whether the apparent spectral broadening reflected a true physiological effect rather than an artifact of intersubject averaging, we quantified the full width at half maximum (FWHM) of the cardiac peak in the power spectrum. The FWHM was significantly greater in ventral CSF than in the vertebral and internal carotid artery spectra, with a similar trend relative to the internal jugular vein spectrum (paired t-tests on log-transformed FWHM: vertebral artery, *p* < 0.001; internal carotid artery, *p* = 0.012; internal jugular vein, *p* = 0.089; Fig. 2C). Furthermore, respiratory power in the ventral CSF was significantly higher than in the vertebral and carotid arteries (*p*_FDR_ < 0.001), with a similar trend relative to the internal jugular vein (*p*_FDR_ = 0.07), indicating that respiration exerts a greater influence on venous and CSF dynamics than on arterial pulsatility (Fig. 2B).

**Fig. 3.**
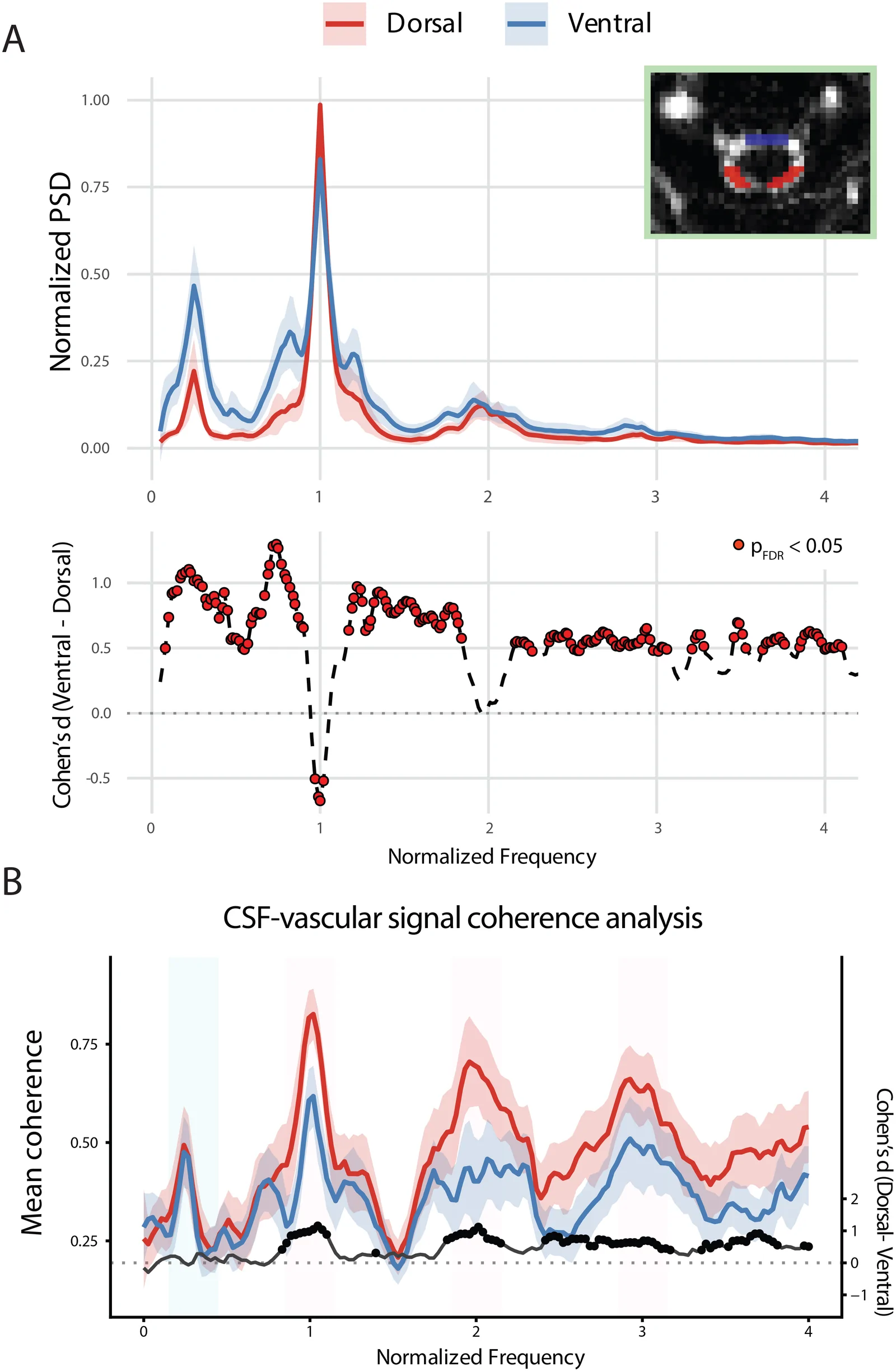
Frequency-dependent differences between ventral and dorsal spinal CSF flow dynamics at C2–C3. (A) *Top*, group-averaged normalized power spectral density (PSD) of ultrafast EPI signals from ventral and dorsal CSF. Ventral CSF exhibited greater power at the respiratory frequency and in the flanks surrounding the cardiac fundamental frequency, whereas dorsal CSF exhibited greater power at the cardiac fundamental. The *inset* illustrates the ventral and dorsal CSF regions of interest. *Bottom*, frequency-resolved Cohen’s d effect sizes for ventral relative to dorsal CSF. Positive values indicate greater power in ventral CSF, and colored markers indicate frequencies surviving false-discovery-rate (FDR) correction. (B) *Top*, mean coherence between CSF and vascular signals for the ventral and dorsal CSF compartments as a function of frequency. Dorsal CSF generally exhibited greater vascular coherence, particularly at the cardiac fundamental and harmonic frequencies. *Bottom*, frequency-resolved Cohen’s d effect sizes for ventral relative to dorsal CSF. Negative values indicate greater coherence in dorsal CSF, and black markers indicate frequencies surviving FDR correction. To account for interindividual variation in respiratory and heart rates, frequencies were rescaled such that the cardiac fundamental frequency equaled 1 and the respiratory frequency was set to 0.25.

In a subset of participants (n = 22) in whom the spinal cord was not abutting the dorsal spinal canal wall, allowing a discernible dorsal CSF column, spectral comparisons were conducted between ventral and dorsal CSF (Fig. 3A). Dorsal CSF exhibited lower power across broad portions of the spectrum, particularly at the respiratory frequency (*p*_FDR_ = 0.002) and on both flanks of the cardiac fundamental frequency (low-frequency flank: *p*_FDR_ < 0.001; high-frequency flank: *p*_FDR_ = 0.002). Conversely, the dorsal column exhibited greater power at the cardiac fundamental frequency (*p*_FDR_ = 0.014).

### 3.3 Neurovascular and spinal CSF flow dynamics preferentially couple at cardiac frequencies

We then examined coupling between the jugular, vertebral, and carotid vascular and CSF signals flow using coherence analysis. Across all vascular signals, coherence with ventral and dorsal spinal canal CSF flow signal was consistently higher at the fundamental cardiac frequency than at the respiratory frequency (ventral: *p* = 0.004; dorsal: *p* < 0.001). Compared with ventral CSF, dorsal CSF exhibited greater coherence with vascular signals at the cardiac fundamental and its first two harmonics (cardiac fundamental: 0.83 ± 0.03 versus 0.62 ± 0.04; first harmonic: 0.7 ± 0.05 versus 0.4 ± 0.05; second harmonic: 0.63 ± 0.04 versus 0.47 ± 0.05; *p*_FDR_ < 0.05; Fig. 3B).

To determine whether shared global fluctuations, including bulk motion, respiratory B_0_ modulation, or scanner-related drift, could spuriously inflate intercompartmental coherence, we repeated the analysis using the vertebral body as a nominally stationary reference. Coherence with the vertebral-body reference was significantly lower than compartment-specific coherence across the respiratory and cardiac frequency bands and cardiac harmonics (Supplementary Fig. S2). The peaks at the respiratory and cardiac frequencies in the reference ROI may reflect respiratory B_0_ modulation (Verma & Cohen-Adad, 2014), low-level physiological bone marrow signal (Biffar et al., 2010), or minor SENSE unfolding. However, their magnitude remained below that of the compartment-specific coherence.

### 3.4 Agreement between ultrafast EPI cardiac cycle detection and physiological log data

**Fig. 4.**
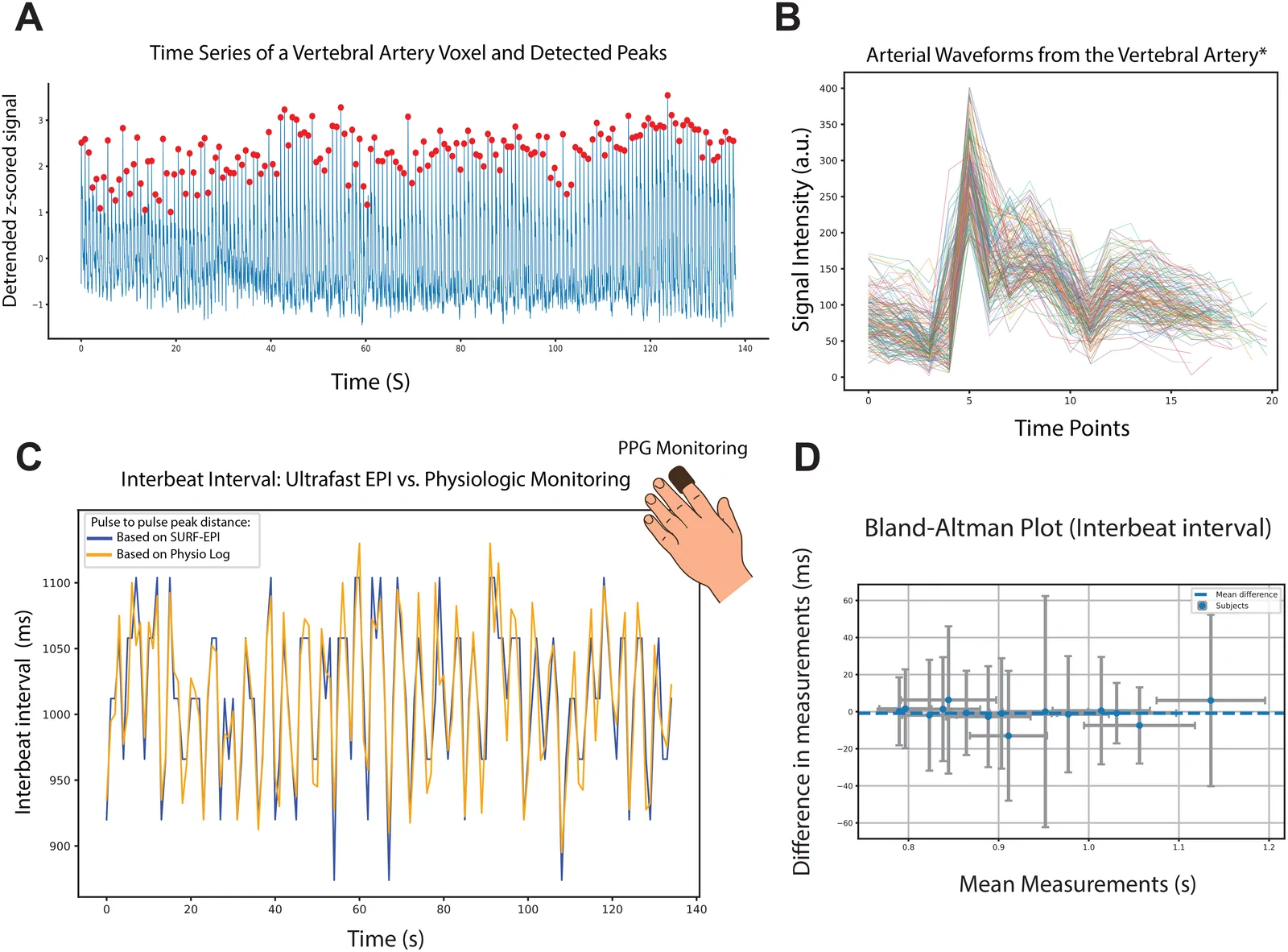
Vascular signal time series enable self-gated cardiac cycle detection in ultrafast EPI. (A) Representative vertebral artery time series with detected systolic peaks used for cardiac cycle identification. (B) Arterial waveforms extracted from the vertebral artery and aligned to detected systolic peaks, illustrating pulse morphology across cycles. (C) Example subject showing beat-to-beat interbeat intervals derived from ultrafast EPI and physiological pulse recordings. (D) Bland–Altman analysis of interbeat intervals across subjects. Each point represents one subject, with the x-axis showing the mean cardiac cycle length and the y-axis showing the mean difference between ultrafast EPI-derived and physiologic interbeat intervals. Vertical and horizontal error bars indicate the standard deviation of interbeat interval differences and cycle lengths, respectively.

Cardiac cycles were identified by applying a peak detection algorithm to the vertebral artery time series (Fig. 4A, B). To evaluate the concordance between cardiac cycle detection from ultrafast EPI and physiological recordings, we calculated the ICC between beat-to-beat cardiac cycle lengths derived from each method (Fig. 4C, D). Mean and proportional bias were negligible, confirming the absence of systematic or magnitude-dependent differences between methods. Among the 15 participants with simultaneous physiological recordings, 93.3% showed either very strong (ICC > 0.80; 33.3%) or strong agreement (ICC = 0.60–0.79; 60.0%). Moderate agreement (ICC = 0.40–0.59) was observed in one of the participants. These results highlight the robustness of ultrafast EPI for concurrent assessment of CSF and vascular dynamics, with cardiac cycle detection closely matching physiologic recordings in most participants.

### 3.5 Alignment of CSF flow waveforms between ultrafast EPI and PC-MRI

**Fig. 5.**
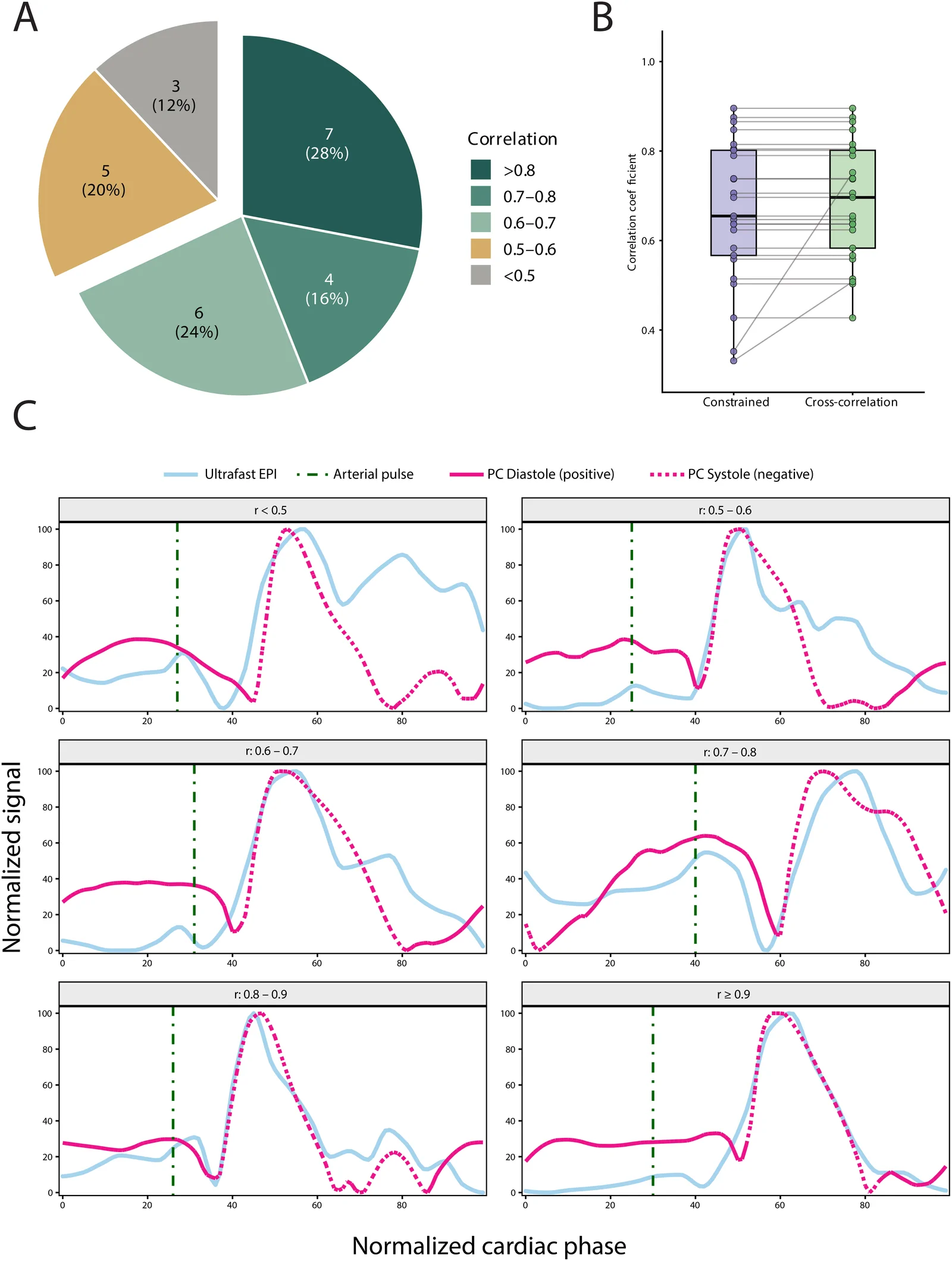
Agreement between ultrafast EPI and phase-contrast MRI CSF waveforms. (A) Distribution of participant-level correlation coefficients between cardiac-cycle waveforms derived from ultrafast EPI and phase-contrast MRI (PC-MRI; *n* = 25), grouped by correlation strength. (B) Paired comparison of correlation coefficients obtained using the prespecified constrained alignment and unconstrained cross-correlation. For the constrained analysis, the PC-MRI systolic CSF peak was restricted to occur between the arterial systolic peak and 35% of the cardiac cycle thereafter. Lines connect measurements from the same participant. (C) Representative ultrafast EPI and PC-MRI waveforms across correlation ranges. Ultrafast EPI signal is shown in light blue, and the arterial systolic peak is indicated by the green dash-dotted line. Positive diastolic and negative systolic portions of the PC-MRI velocity waveform are shown by solid and dotted magenta lines, respectively. Waveforms were normalized to their maximum and are displayed across the normalized cardiac cycle.

CSF flow waveforms were extracted from a ventral spinal canal ROI at the C2–C3 level in both ultrafast EPI and PC-MRI datasets (Supplementary Fig. S3). To enable direct comparison, ultrafast EPI signals were up-sampled to match the number of cardiac phases in the phase-contrast MRI, and PC-MRI waveforms were converted to absolute values to account for the non-directionality of ultrafast EPI. PC-MRI–ultrafast-EPI cross-correlation was constrained such that the PC-MRI systolic CSF peak occurred between the arterial systolic peak and 35% of the cardiac cycle length thereafter. Waveform similarity was assessed using correlation analysis. In 68% of participants, either very strong (r ≥ 0.80; n = 7; 28.0%) or strong (r = 0.60–0.80; n = 10; 40%) correlation was observed between waveforms from the two modalities (Fig. 5). Five participants (20%) showed moderate correlations (r = 0.5–0.6), whereas the remaining three (12%) showed weak correlations (r < 0.50). In an unconstrained cross-correlation sensitivity analysis, correlation increased in only 2 of 25 participants (Fig. 5B), indicating that waveform alignment was largely determined by intrinsic waveform shape rather than the imposed temporal constraint.

### 3.6 Dorsal spinal CSF flow demonstrates lower cycle-to-cycle variability

**Fig. 6.**
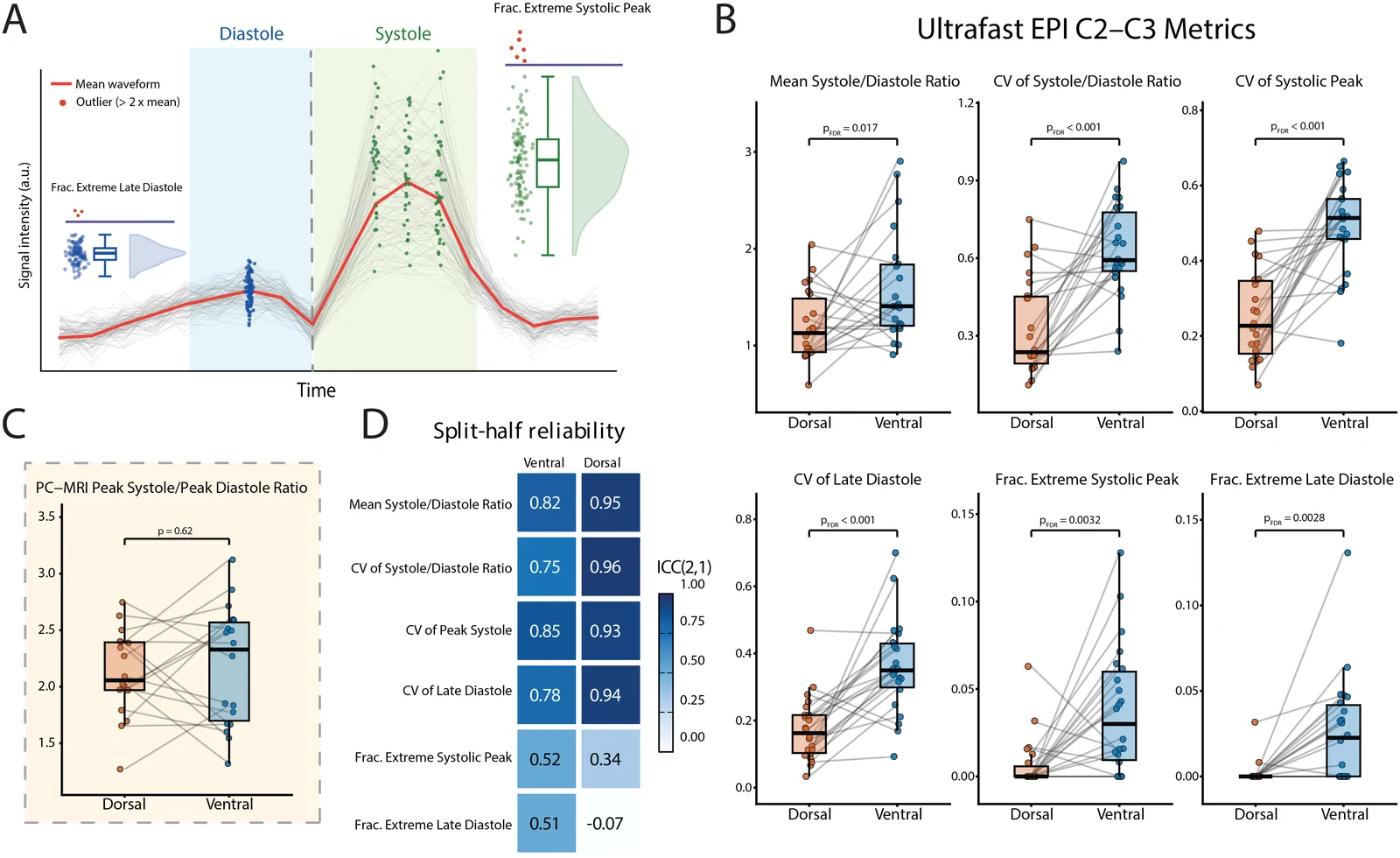
Ventral–dorsal differences in cardiac-cycle variability and systolic–diastolic signal dynamics at C2–C3. (A) Illustration of waveform-derived metrics derived from ultrafast EPI. The mean systole-to-diastole ratio was defined as the peak systolic signal divided by the mean late-diastolic signal. Beat-to-beat variability was quantified using the coefficients of variation (CVs) of peak systolic signal, late-diastolic signal, and the systole-to-diastole ratio. Extreme cycles were defined as those in which the peak systolic or late-diastolic signal exceeded twice the participant-specific mean. Gray traces represent individual cardiac cycles, and the red trace represents the mean waveform. (B) Paired comparisons between dorsal and ventral CSF. Ventral CSF exhibited a higher mean systole-to-diastole ratio, greater CVs for all three waveform measures, and higher fractions of cardiac cycles with extreme peak-systolic and late-diastolic signals. Reported *p* values were corrected for multiple comparisons using the false discovery rate (FDR). (C) The PC-MRI–derived peak-systole-to-peak-diastole velocity ratio did not differ between dorsal and ventral CSF. (D) Split-half reliability of the ultrafast EPI-derived metrics, quantified using intraclass correlation coefficients (ICC(2,1)) separately for ventral and dorsal CSF.

Ultrafast EPI not only reproduced the average CSF waveform across the cardiac cycle (analogous to phase-contrast MRI) but also enabled quantification of beat-to-beat variability. Metrics included the systole-to-diastole signal ratio, defined as peak-systolic signal divided by mean late-diastolic signal; the across-cycle coefficients of variation for peak-systolic signal, late-diastolic signal, and the systole-to-diastole ratio; the proportions of cycles in which peak-systolic or late-diastolic signal exceeded twice the corresponding mean (Fig. 6A). Split-half reliability was good to excellent for the systole-to-diastole ratio and all coefficients of variation across the ventral and dorsal CSF compartments (ICC(2,1) = 0.75–0.96). The proportions of cardiac cycles with extreme peak systole and late-diastolic signal showed moderate reliability in ventral CSF (ICC(2,1) = 0.51–0.52) but poor reliability in dorsal CSF (ICC(2,1) = −0.07–0.34), likely reflecting the rarity of these events and the greater sampling variability introduced by dividing the acquisition into two equal segments (Fig. 6D; Supplementary Fig. S4).

We next compared these waveform-derived measures between the ventral and dorsal spinal CSF columns at C2–C3 (Fig. 6). Ventral CSF exhibited significantly greater coefficients of variation for peak systole, late diastole, and the systole-to-diastole ratio (all *p_FDR_* < 0.001), as well as higher proportions of cardiac cycles with extreme peak-systolic (p_FDR_ = 0.003) and late-diastolic signals (*p_FDR_* = 0.003). Ventral CSF also demonstrated a significantly higher mean systole-to-diastole ratio (*p_FDR_* = 0.017). We examined whether these regional differences persisted after accounting for ROI-related measurement factors. The mean systolic-to-diastolic signal ratio did not differ between ventral and dorsal CSF after adjustment for tSNR or tCNR, although it was higher in ventral CSF after adjustment for ROI size (p = 0.02; Supplementary Table S1). By contrast, the coefficients of variation for peak systole, late diastole, and the systolic-to-diastolic ratio remained higher in ventral CSF after adjustment for tSNR, tCNR, or ROI size (all p ≤ 0.003). The fraction of extreme systolic cycles also remained higher in ventral CSF across all adjusted models, whereas the difference in extreme late-diastolic cycles remained significant after adjustment for tCNR or ROI size, but not tSNR. Finally, the PC-MRI–derived peak-systole-to-peak-diastole ratio did not differ between ventral and dorsal CSF, consistent with the distinct contrast mechanisms of velocity-encoded and inflow-weighted measurements.

### 3.7 Regional signatures of spinal CSF dynamics are reproduced at the craniocervical junction

**Fig. 7.**
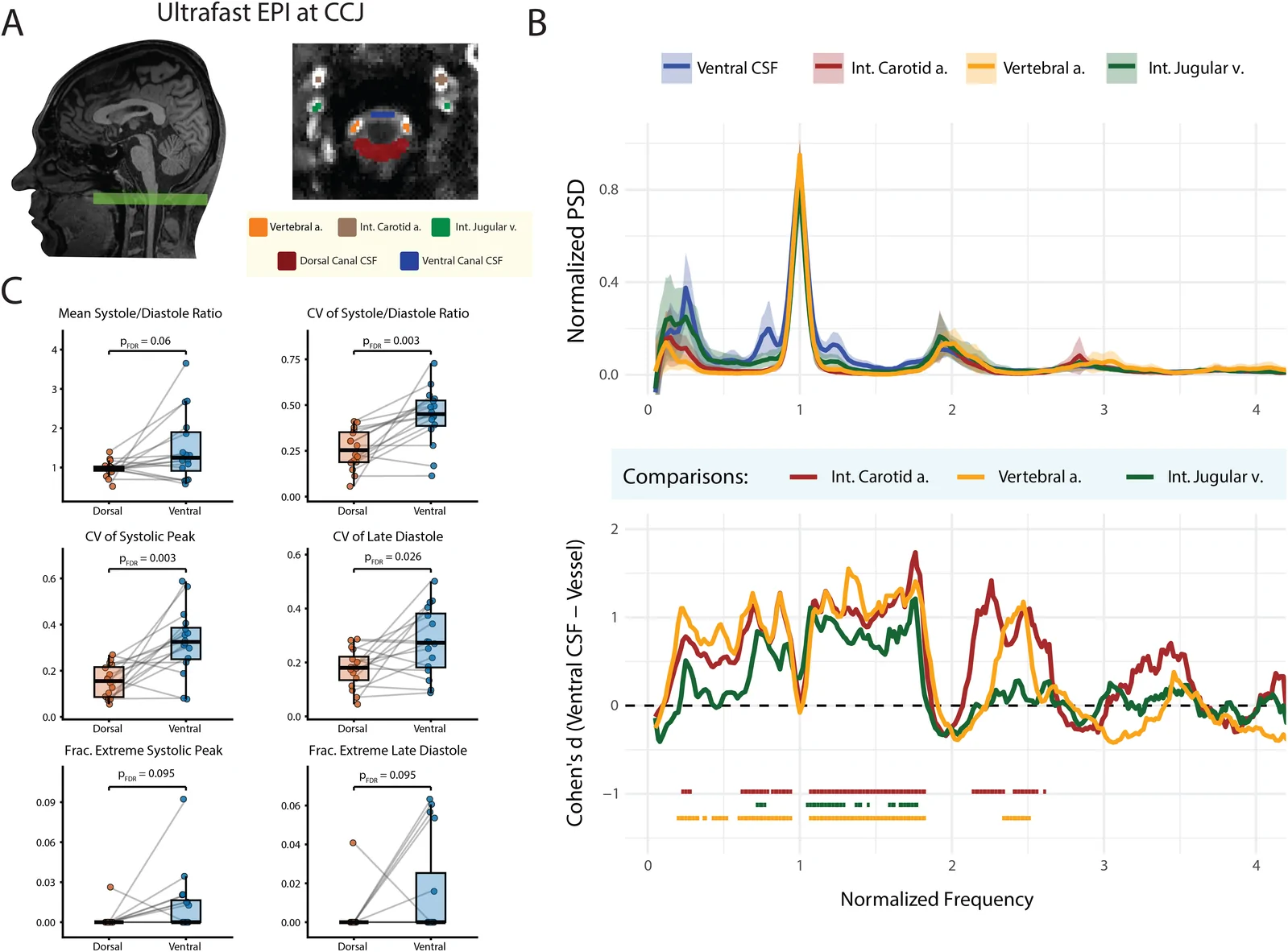
Ultrafast inflow-weighted EPI at the craniocervical junction (CCJ). (A) Sagittal anatomical image illustrating placement of the ultrafast EPI slice at the CCJ, immediately inferior to the foramen magnum, and representative axial ultrafast EPI temporal strand deviation map showing the ventral and dorsal CSF and vascular regions of interest. (B) *Top*, group-averaged normalized power spectral density (PSD) of CCJ signals from ventral spinal canal CSF, internal carotid arteries, vertebral arteries, and internal jugular veins (n = 16). Frequencies were normalized such that the cardiac fundamental was set to 1 and the respiratory frequency to 0.25. Shaded bands indicate 95% confidence interval. *Bottom*, frequency-resolved Cohen’s *d* for ventral CSF relative to each vascular compartment; positive values indicate greater spectral power in ventral CSF. Colored markers denote frequencies surviving false-discovery-rate (FDR) correction. (C) Paired comparisons of waveform-derived metrics between dorsal and ventral CSF. Metrics included the mean systole-to-diastole signal ratio, defined as the peak systolic signal divided by the mean late-diastolic signal; the across-cycle coefficient of variation (CV) of this ratio; the CVs of peak systolic and late-diastolic signals; and the fractions of cardiac cycles exhibiting extreme systolic or late-diastolic fluctuations, defined as signals exceeding twice the participant-specific mean. Lines connect measurements from the same participant; reported *p* values are FDR corrected.

To test whether the CSF dynamics observed at C2–C3 were reproduced at the craniocervical junction (CCJ), we repeated the spectral and cycle-to-cycle analyses using a separate acquisition obtained immediately below the foramen magnum (n = 16; Fig. 7). Consistent with the findings at C2–C3, the ventral CSF power spectral density at the CCJ exhibited prominent side peaks flanking the fundamental cardiac frequency, with point-wise comparisons showing significantly greater power at these frequencies than in all vascular ROIs (p_FDR_ < 0.05). Respiratory-frequency power was higher in ventral CSF than in the vertebral and carotid arteries (p_FDR_ < 0.05), but did not differ from that in the internal jugular vein. For the ventral–dorsal comparison, the CCJ showed a pattern similar to C2–C3, with uncorrected point-wise trends toward greater ventral power at low frequencies and at frequencies flanking the cardiac fundamental frequency (Supplementary Fig. S5). Cycle-to-cycle analyses showed a similar ventral–dorsal pattern at the CCJ, with higher coefficients of variation in ventral CSF for peak systole, late diastole, and the systole/diastole ratio (all p_FDR_ < 0.05). The mean systole/diastole ratio and the proportions of cycles with extreme systolic peaks or late-diastolic values were also numerically higher ventrally, but did not reach significance (p_FDR_ = 0.06–0.095).

### 3.8 Exploratory characterization of TR effects on CSF and vascular signal

**Fig. 8.**
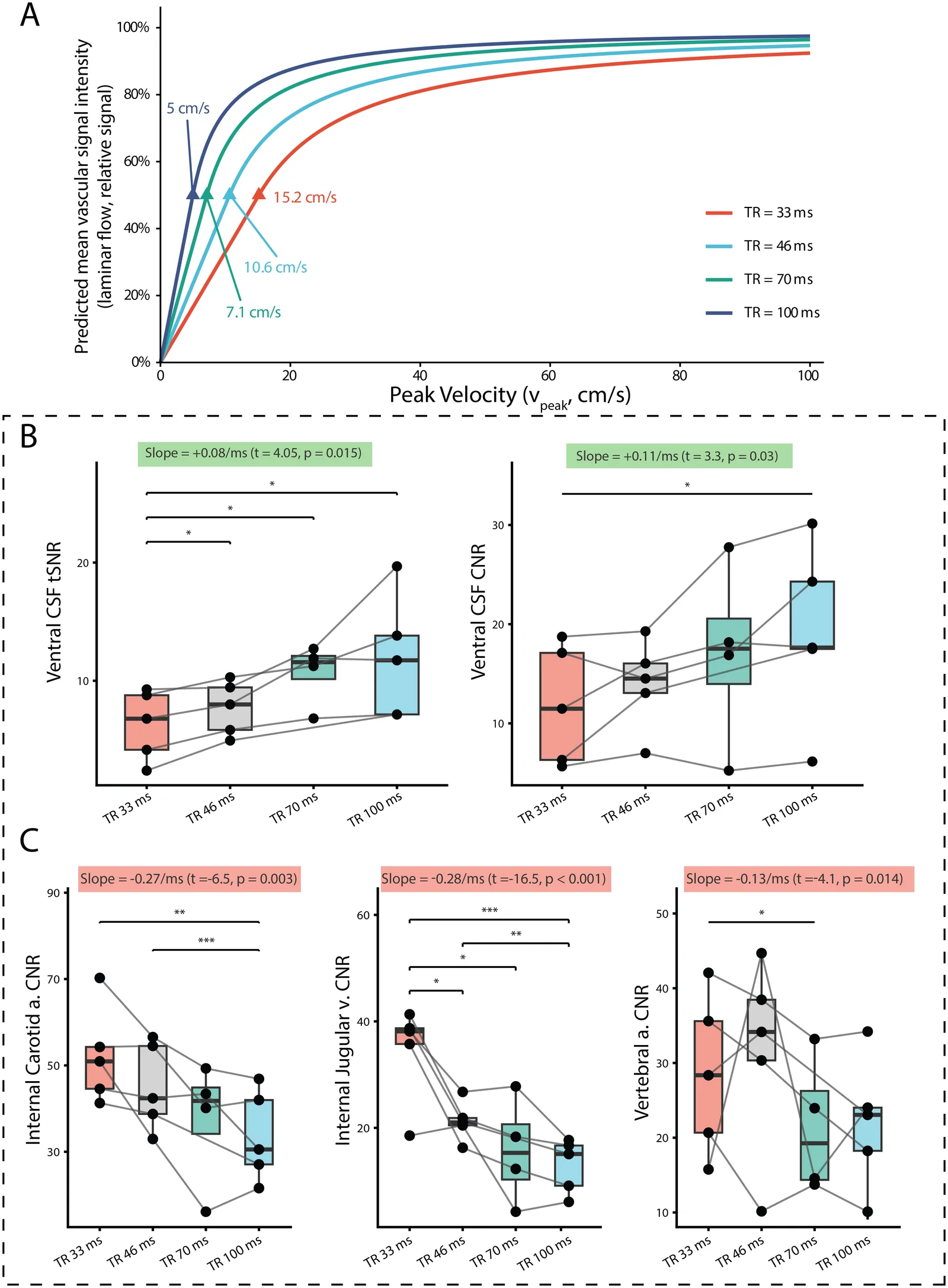
Repetition time modulates the inflow-sensitive dynamic range of ultrafast EPI. (A) Theoretical relationship between mean inflow-weighted vascular signal and peak velocity for unidirectional laminar flow, assuming a 90° flip angle, a 5-mm slice thickness, and repetition times (TRs) of 33, 46, 70, and 100 ms. For each TR, signal increased approximately linearly with velocity up to the critical velocity for complete spin replacement, v max = Δz/TR, and then increased sublinearly toward saturation. Triangles indicate v max for each TR (15.2, 10.6, 7.1, and 5.0 cm/s, respectively). (B) Ventral CSF temporal signal-to-noise ratio (tSNR; left) and temporal contrast-to-noise ratio (tCNR; right) across TRs in the exploratory multi-TR cohort (n = 5). Both measures increased with longer TR. (C) Vascular tCNR across TRs for the internal carotid arteries (left), internal jugular veins (middle), and vertebral arteries (right), demonstrating decreasing vascular tCNR with increasing TR. Monotonic dependence on TR was assessed by fitting an ordinary least-squares regression for each participant and testing the resulting participant-specific slopes against zero using a one-sample *t*-test. Colored annotations report the mean slope, *t* statistic, and *p* value. Brackets indicate significant pair-wise comparisons (uncorrected *p < 0.05, **p < 0.01 and **p < 0.001).

Theoretical predictions of the relationship between mean inflow-weighted signal and peak velocity for unidirectional laminar vascular flow were generated for a 90° flip angle, a 5-mm slice thickness, and the TRs used in this study (33, 46, 70, and 100 ms; Fig. 8; Supplementary Fig. S6). For each TR, mean inflow signal increased linearly with peak velocity up to v_max_ = Δz/TR, then increased sublinearly toward saturation. Shorter TRs shifted this transition to higher velocities, preserving greater sensitivity to velocity changes at faster vascular flow.

To assess how repetition time influenced CSF and vascular signal, we performed an exploratory multi-TR analysis in five participants scanned at TRs of 33, 46, 70, and 100 ms. Ventral CSF tSNR increased with TR (mean slope = 0.08/ms, *p* = 0.015), as did ventral CSF tCNR (mean slope = 0.11/ms, *p* = 0.03; Fig. 8). In contrast, vascular tCNR decreased with increasing TR in the internal carotid arteries (slope = −0.272/ms, p = 0.0029), internal jugular veins (slope = −0.282 ms, p < 0.001), and vertebral arteries (slope = −0.132/ms, P = 0.014; Fig. 8). The greater vascular tCNR with shorter TR is consistent with a higher v_max_ (Δz/TR) and a broader inflow-sensitive dynamic range.

## 4 Discussion

Our findings establish ultrafast EPI as a simple, robust method for capturing real-time CSF and vascular dynamics simultaneously without the constraints of velocity encoding or external gating. While the average CSF waveform derived from ultrafast EPI generally paralleled PC-MRI, the method also enabled spectral decomposition and cycle-to-cycle variability analysis that extend beyond what conventional PC-MRI provides. In this sense, ultrafast EPI offers not only an acquisition strategy but also an analytic framework for probing CSF flow in both the time and frequency domains.

Similar to real-time PC-MRI findings (Laganà et al., 2022), respiratory contributions were more prominent in the ventral CSF and internal jugular veins than in the carotid or vertebral arteries. During inspiration, thoracic expansion lowers intrathoracic pressure, increasing the pressure gradient between the heart and extrathoracic veins and thereby augmenting venous return (Laganà et al., 2017; Zamboni et al., 2012). At the cervical spine, enhanced cranial venous drainage during inspiration is commonly accompanied by rostral CSF displacement, helping maintain intracranial volume, particularly during deep breathing (Dreha-Kulaczewski et al., 2015). However, the direction of CSF movement also depends on the transmission of thoraco-abdominal pressure through the epidural venous plexus, which can drive caudal cervical CSF displacement under some conditions (Lloyd et al., 2020). Overall, these support a shared respiratory modulation of the venous–CSF system.

In the ventral CSF, we observed spectral widening around the fundamental cardiac frequency, a feature that was absent in vascular signals. This broadening may reflect the viscoelastic and compliance properties of the intracranial system, which is in communication with the ventral spinal subarachnoid space (Egnor, 2019; Wagshul et al., 2011). Prior studies suggest that the cranium normally functions as a pulsation absorber, acting like a notch filter that attenuates the fundamental cardiac frequency (Zou et al., 2008). Another explanation is that these represent sidebands generated by respiratory modulation of the cardiac pulsation, a phenomenon recently characterized with magnetic resonance encephalography (MREG) as cardiorespiratory envelope modulation (Kiviniemi et al., 2025; Raitamaa et al., 2021). Nevertheless, differences in flow velocity and signal-to-noise characteristics between vascular and CSF signals may also contribute to the broader CSF spectrum and should be considered when interpreting this finding.

Compared with the dorsal spinal CSF column, the ventral canal exhibited a more complex spectral profile, with greater respiratory and non-cardiac contributions, higher beat-to-beat variability, and lower coupling with neurovascular flow. This divergence likely reflects anatomical and functional differences between the compartments, as the spinal subarachnoid space is structurally compartmentalized by arachnoid membranes anchored to the denticulate ligaments and nerve rootlets, resulting in distinct ventral and dorsal cisternal spaces (Dauleac et al., 2019; Pahlavian et al., 2014). Furthermore, the ventral CSF space serves as the primary conduit between the spinal canal and the cranial vault (Yiallourou et al., 2012). Consistent with this, time-spatial labeling inversion pulse (time-SLIP) imaging has shown that respiration drives substantial bidirectional motion in the prepontine cistern, with cephalad displacement during inspiration and caudad displacement during expiration (Yamada et al., 2013). Additional factors may contribute to the observed compartmental differences, including posture-dependent displacement of the spinal cord within the thecal sac during supine imaging, which effectively enlarges the ventral subarachnoid space, and differences in vascular anatomy, with a single anterior spinal artery ventrally and paired posterior spinal arteries dorsolaterally, potentially shaping local pulsatility and CSF–vascular coupling.

Spectral analysis has been used with inflow-weighted imaging to probe low-frequency CSF flow fluctuations (Ashenagar et al., 2025; Fultz et al., 2019). Yet, limited sampling rates (2–5 Hz) constrained prior studies to slow oscillatory components. Ultrafast EPI, with effective sampling rates exceeding 20 Hz, resolves higher-frequency content, including the cardiac fundamental and its harmonics. Related work with MREG has demonstrated whole-brain sampling at ~10 Hz by employing strong k-space undersampling (Kiviniemi et al., 2025). However, MREG is fundamentally a T2*-weighted blood oxygen level dependent (BOLD) technique, yielding a complex composite of vascular, brain tissue, and CSF signals at relatively low spatial resolution. Thus, while valuable for mapping global brain pulsations, MREG cannot isolate CSF flow from vascular dynamics. At the highest temporal resolution, pencil-beam imaging samples up to ~40 Hz, yet its one-dimensional acquisition sacrifices spatial mapping and cannot simultaneously assess blood and CSF signals (Bhadelia et al., 2013; Zhao et al., 2016).

Rapid imaging of cervical arteries enables extraction of vessel-specific blood flow waveforms that can be used for self-gating. Arterial waveforms derived directly from ultrafast EPI may more closely reflect blood inflow toward the brain and its temporal relationship with CSF motion than peripheral pulse recordings. In PC-MRI, cardiac gating often relies on fingertip photoplethysmography, which is subject to variable time lags (pulse arrival time) influenced by factors such as hypertension, owing to the delay between cardiac contraction and peripheral pulse detection (Finnegan et al., 2021). While EKG gating offers more direct cardiac timing and minimizes temporal variability in CSF flow measurements, its practical challenges (e.g., the need for chest shaving to ensure reliable skin contact) often restrict its use to smaller studies (Bert et al., 2020; Nacif et al., 2012). By contrast, simultaneous imaging of neurovascular and CSF flow enables precise temporal characterization of CSF dynamics with respect to the cerebral arterial input function, avoiding the limitations of peripheral gating. Furthermore, ultrafast inflow-weighted EPI may have translational implications for neurovascular disorders that alter arterial waveform morphology or timing, such as carotid steno-occlusive disease and intracranial hemorrhage (Demchuk et al., 2001; MacIntosh et al., 2011; Mayer et al., 1996; Tahmasebpour et al., 2005). By simultaneously sampling cervical arterial inflow and CSF motion, ultrafast EPI may help determine whether delayed, dampened, or otherwise distorted arterial waveforms affect neurovascular–CSF coupling.

The mean CSF flow waveform derived from ultrafast EPI showed strong correlation with PC-MRI across the cardiac cycle in most subjects, although in some individuals the correlation was only moderate or even weak. This discrepancy likely reflects fundamental differences in contrast mechanisms between ultrafast inflow-weighted EPI and PC-MRI. While PC-MRI provides quantitative directional velocity measurements, CSF signal dynamics on inflow-weighted EPI reflect a nonlinear, history-dependent magnitude enhancement generated by the entry of relatively unsaturated spins (Ashenagar et al., 2025; Diorio et al., 2024; Kim et al., 2022). Relating the two measurements may, therefore, require signal modeling that accounts for their distinct contrast mechanisms, as a simple linear model may not fully capture their relationship. Furthermore, flow reversals may produce oppositely directed velocities at the same cardiac phase across different cardiac cycles, which can cancel during cardiac-gated averaging and yield a net velocity near zero. In contrast, ultrafast EPI is sensitive to inflow magnitude irrespective of the direction, such that oppositely directed flow contributes to signal enhancement rather than cancellation. This distinction may be particularly important during late diastole, when respiratory modulation can induce craniocaudal flow reversals, whereas its impact may be more limited during early systole, when the caudal flow direction remains relatively unaffected (Liu et al., 2024). Taken together, ultrafast EPI can complement PC-MRI by enabling time-resolved assessment of CSF dynamics at high temporal resolutions, while PC-MRI provides directional and quantitative velocity estimates. However, rather than a direct instantaneous mapping, calibration would likely require modeling the ultrafast EPI signal as a temporally integrated inflow process (Ashenagar et al., 2025), potentially leveraging the full time series and incorporating velocity estimates from PC-MRI to constrain or learn the signal–velocity relationship. Future work is needed to develop subject-specific calibration frameworks, particularly during phases of the cardiac cycle with consistent flow direction (e.g., early systole).

Real-time PC-MRI provides quantitative velocity and directionality at temporal resolutions >10 Hz, enabling characterization of cardiac, respiratory, and low-frequency physiologic components (Chen et al., 2015; Laganà et al., 2022; Liu et al., 2024, 2025; Yildiz et al., 2017; Zhu et al., 2025). The distinction of ultrafast EPI is that it trades directional velocity encoding for a faster, simpler, and more broadly deployable inflow-weighted acquisition that captures CSF and vascular signal dynamics within the same sequence. Ultrafast EPI relies on a standard inflow-weighted EPI readout rather than a specialized real-time PC-MRI implementation, making it broadly deployable across research and clinical MRI platforms. In addition, the absence of velocity-encoding gradients permits very high temporal resolution; in the present study, this enabled sampling rates at >20–30 Hz with spatial resolution comparable with real-time PC-MRI. This temporal efficiency could instead be traded for higher in-plane spatial resolution or extend coverage to additional slices, with further gains in coverage achievable through simultaneous multislice acceleration (Barth et al., 2016; Chen et al., 2015; Setsompop et al., 2012), while preserving temporal resolution comparable with real-time PC-MRI.

Another advantage of ultrafast EPI over real-time PC-MRI is its broad effective dynamic range. In PC-MRI, selection of a single VENC requires a trade-off between sensitivity to slow CSF motion and accurate encoding of faster vascular flow (Liu et al., 2025). A low VENC improves sensitivity to CSF velocity but increases susceptibility to vascular aliasing, whereas a high VENC permits measurement of faster blood flow at the expense of lower SNR for CSF motion. Ultrafast EPI, by comparison, is sensitive to inflow across a broader range of velocities, enabling simultaneous characterization of CSF and vascular waveforms and their temporal coupling. This sensitivity is nevertheless bounded by the critical through-plane velocity required for complete spin replacement within each TR (v_max_ = Δz/TR) (Diorio et al., 2024; Gao et al., 1988). As peak vascular velocity exceeds v_max_, the inflow-weighted signal becomes progressively sublinear and approaches saturation. Thus, although vascular systolic and diastolic phases remain clearly distinguishable, ultrafast EPI progressively becomes less sensitive to small changes at high-flow velocities. The broader effective range of ultrafast EPI may be relevant for capturing transient CSF fluctuations and task-based imaging. Even under resting conditions, beat-to-beat analysis showed that CSF flow can exceed twice the average peak amplitude in up to 13% of cycles, fluctuations that may introduce aliasing and measurement errors in PC-MRI but are captured by ultrafast EPI. Given that each ultrafast EPI frame is fully sampled, rather than reconstructed from k-space sub-sampling with k-space-time acceleration (Dreha-Kulaczewski et al., 2015; Lin et al., 2012; Yildiz et al., 2017), the method achieves true high temporal resolution without temporal blurring, while maintaining high in-plane spatial resolution.

A limitation of ultrafast EPI is that it provides a non-directional inflow-weighted CSF signal with cranial and caudal motion both appearing as positive signal changes. This differs from real-time PC-MRI, in which cranial and caudal CSF motion are resolved as velocities with opposite phase signs. Therefore, caution is needed when comparing ultrafast EPI with direction-encoded real-time PC-MRI in frequency-domain analyses, as the non-directional ultrafast EPI waveform may alter the apparent harmonic content of CSF motion. Similarly, systolic and diastolic phases of CSF flow in ultrafast EPI were defined by their temporal relationship with the simultaneously imaged arterial waveform, rather than by the sign of CSF velocity as in real-time PC-MRI. Future studies with contiguous multislice ultrafast EPI implementations may help infer flow directionality by tracking the craniocaudal progression of inflow-related signal changes across slices, an approach established in prior inflow-weighted CSF dynamics work (Fultz et al., 2019) and recently extended to bidirectional CSF flow imaging (Yang et al., 2025). The absence of a ventral–dorsal difference in the PC-MRI-derived peak-systole-to-peak-diastole ratio, despite differences observed with ultrafast EPI, may reflect differences in the contrast mechanisms and acquisition strategies of the two methods rather than a true discrepancy in the underlying CSF dynamics. Future studies directly comparing real-time PC-MRI with ultrafast EPI will be needed to determine whether the observed ventral–dorsal differences represent genuine physiological differences or arise from methodological factors.

Several additional limitations warrant consideration. First, our analyses were rescaled to cardiac and respiratory frequencies, and thus other sources of very low-frequency variance may not have been fully captured, as they are not necessarily coupled to these frequencies. Second, CSF dynamics were quantified using ventral and dorsal subarachnoid space ROIs, which may mask finer-scale spatial heterogeneity. Future voxel-wise approaches could resolve spatiotemporal CSF flow patterns beyond ROI-level analyses. Third, the present implementation of ultrafast EPI was limited to single-slice acquisitions; however, simultaneous multislice imaging could enable concurrent assessment of spinal and intracranial CSF flow without significant time penalty (Barth et al., 2016). Fourth, our study was restricted to healthy subjects, and future investigations in patient populations will be essential to determine how alterations in CSF dynamics manifest across time and frequency domains and how they relate to intracranial pathophysiology, including structural abnormalities, disturbed CSF circulation, and altered viscoelastic properties. Finally, although a frame rate of 20 Hz exceeds the Nyquist limit even for cardiac harmonics, precise evaluation of phase shifts and characterization of vascular and CSF waveforms benefit from even higher temporal resolution. To this end, advanced undersampling and acceleration strategies could be leveraged to achieve higher sampling rates (Uecker et al., 2010).

In conclusion, ultrafast EPI provides a simple and robust means to capture real-time CSF and neurovascular dynamics with high temporal fidelity and a wide dynamic range. By enabling simultaneous assessment of arterial inflow, venous outflow, and CSF motion, ultrafast EPI reveals spectral and cardiac cycle-resolved features that conventional PC-MRI cannot capture. More broadly, the correspondence between ultrafast EPI and PC-MRI waveforms suggests that quantitative velocity measurements from PC-MRI could be combined with the fast, non-directional dynamics captured by ultrafast EPI to yield unified, calibratable markers of CSF flow in real time. Beyond cardiac-cycle–locked dynamics, the real-time nature of ultrafast EPI provides a framework for probing off–cardiac-cycle CSF flow patterns and task-mediated perturbations, such as Valsalva maneuvers or coughing, enabling interrogation of transient, physiologically evoked CSF responses. By providing a simple, robust, and broadly deployable method for real-time CSF imaging, ultrafast EPI enables investigation of CSF flow dynamics across health and disease and lays a foundation for future applications spanning intracranial pressure–volume disorders, hydrocephalus, CSF flow obstruction, and neurovascular disorders

## Supplementary Material

Supplementary Material

## Data Availability

All relevant code developed for this study is publicly available (https://github.com/BRAFTI/surf-epi). Imaging data supporting the findings of this study are available from the corresponding author upon reasonable request.
