# Supplementary material for: Simultaneous real-time imaging of upper cervical cerebrospinal fluid and vascular dynamics using ultrafast inflow-weighted EPI
Source: Imaging Neurosci (Camb). 2026 Sep 22;4:IMAG.a.1374. doi: 10.1162/IMAG.a.1374 (PMC13600270; doi:10.1162/IMAG.a.1374)
Supplement: Supplementary file 1 [file IMAG.a.1374_supp.pdf]

Supplementary Information – Simultaneous Real-time Imaging of Upper Cervical Cerebrospinal Fluid and Vascular Dynamics Using Ultrafast Inflow-weighted EPI

Amir H. Shaker, Amir H. G. Arani, Bryn A. Martin, Aristeidis Sotiras, Cihat Eldeniz, Helia Hosseini, Joshua S. Shimony, David D. Limbrick Jr., Jennifer M. Strahle, Arash Nazeri*

*Corresponding Author: a.nazeri@wustl.edu

Outcome measure	tSNR	tCNR	ROI size (n voxels)
**Mean systolic to diastolic ratio**	t = -0.7, p = 0.46	t = -1.9, p = 0.06	t = -2.5, p = 0.02
**CV of systolic to diastolic ratio**	t = -3.8, p < 0.001	t = -5.0, p < 0.001	t = -5.3, p < 0.001
**CV of peak systole**	t = -4.6, p < 0.001	t = -6.0, p < 0.001	t = -6.4, p < 0.001
**CV of late diastole**	t = -3.1, p = 0.003	t = -4.6, p < 0.001	t = -5.0, p < 0.001
**Frac. of extreme peak systole**	t = -2.3, p = 0.029	t = -3.4, p = 0.001	t = -4.8, p < 0.001
**Frac. of extreme diastole**	t = -1.7, p = 0.09	t = -3.0, p = 0.005	t = -3.9, p < 0.001

Supplementary Table S1. Regional differences in reconstructed CSF signal metrics after adjustment for ROI-related measurement factors. Linear models were fit with region (ventral vs. dorsal CSF) as the primary factor, with separate models adjusting for temporal signal-to-noise ratio (tSNR), temporal contrast-to-noise ratio (tCNR), or ROI size (voxel count). Reported values correspond to t-statistics and associated p-values for the regional effect.

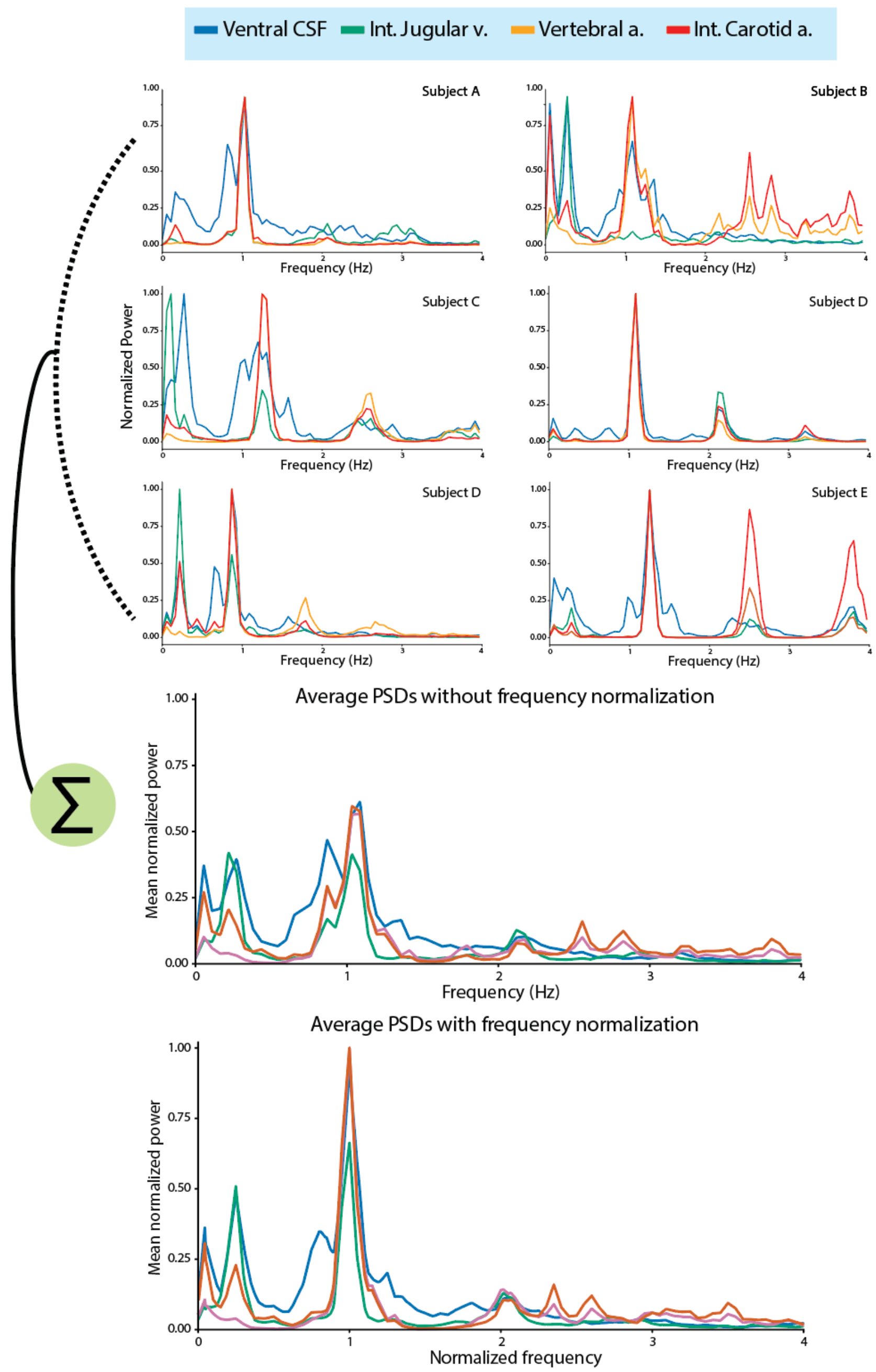

Figure S1. Frequency normalization aligns inter-individual physiological frequencies for population-level spectral averaging. *Top*, normalized power spectral density (PSD) curves from six representative participants for ventral cerebrospinal fluid (CSF), internal jugular vein, vertebral artery and internal carotid artery signals, illustrating inter-individual variability in respiratory and cardiac frequencies. *Middle*, population-averaged PSDs calculated in the original frequency domain (Hz), in which variability in respiratory and heart rates broadens and partially misaligns physiological peaks. *Bottom*, population-averaged PSDs after subject-specific frequency normalization. To account for inter-individual variability in respiratory and heart rates, frequencies were rescaled such that the cardiac fundamental equaled 1 and the respiratory frequency equaled 0.25, thereby aligning physiological peaks across participants before averaging.

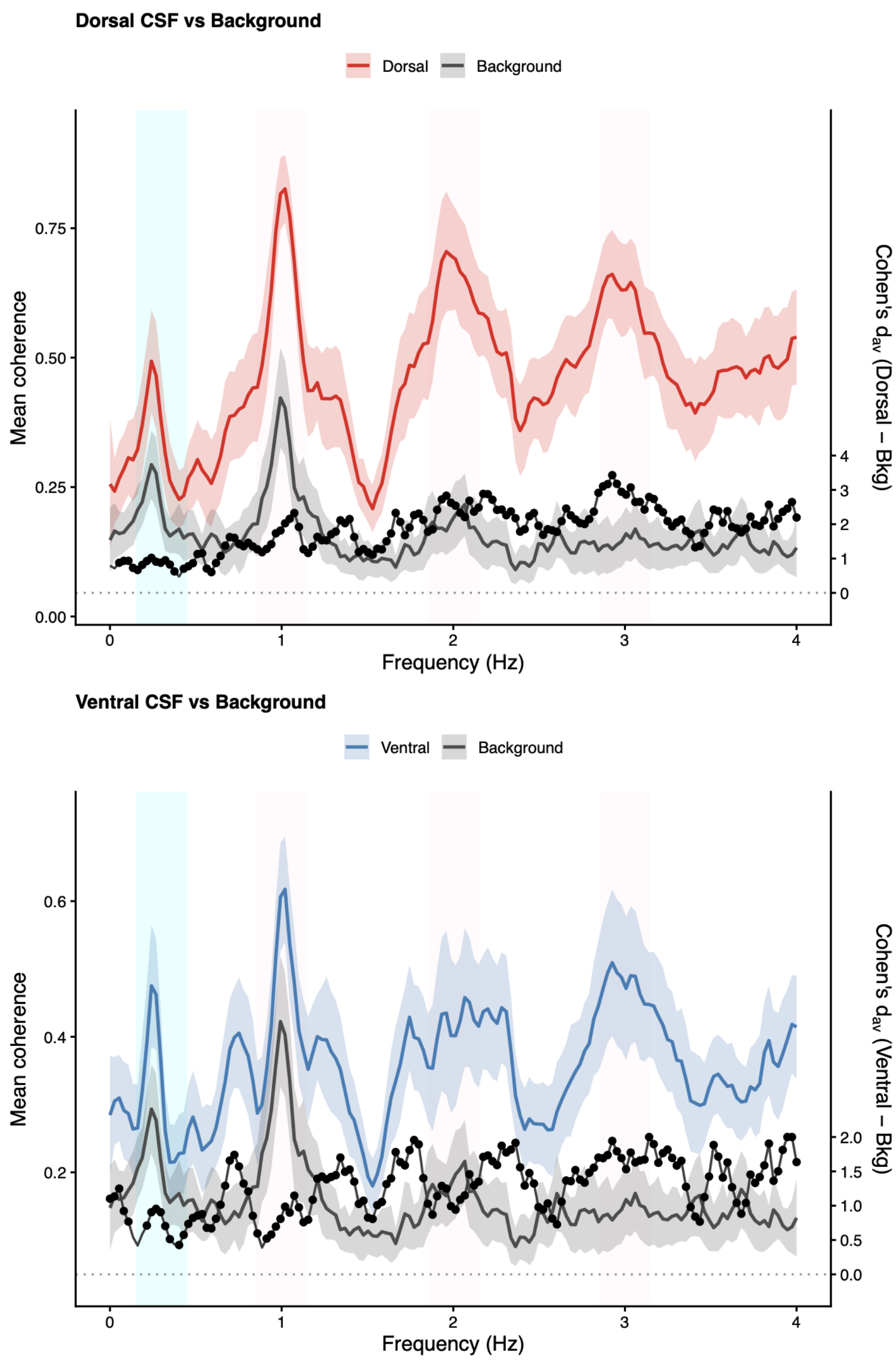

Figure S2. CSF–vascular coherence exceeds coherence with a stationary vertebral-body reference at C2–C3. *Top*, mean magnitude-squared coherence between vascular signals and dorsal CSF or a stationary vertebral-body reference as a function of normalized frequency. *Bottom*, corresponding coherence between vascular signals and ventral CSF or the vertebral-body reference. Coherence with the stationary reference was substantially lower than CSF–vascular coherence across the respiratory and cardiac frequency bands and cardiac harmonics. Black traces show frequency-resolved effect sizes for CSF relative to the vertebral-body reference, with dots indicating frequencies surviving false-discovery-rate correction. Frequencies were rescaled such that the cardiac fundamental frequency equaled 1 and the respiratory frequency was set to 0.25.

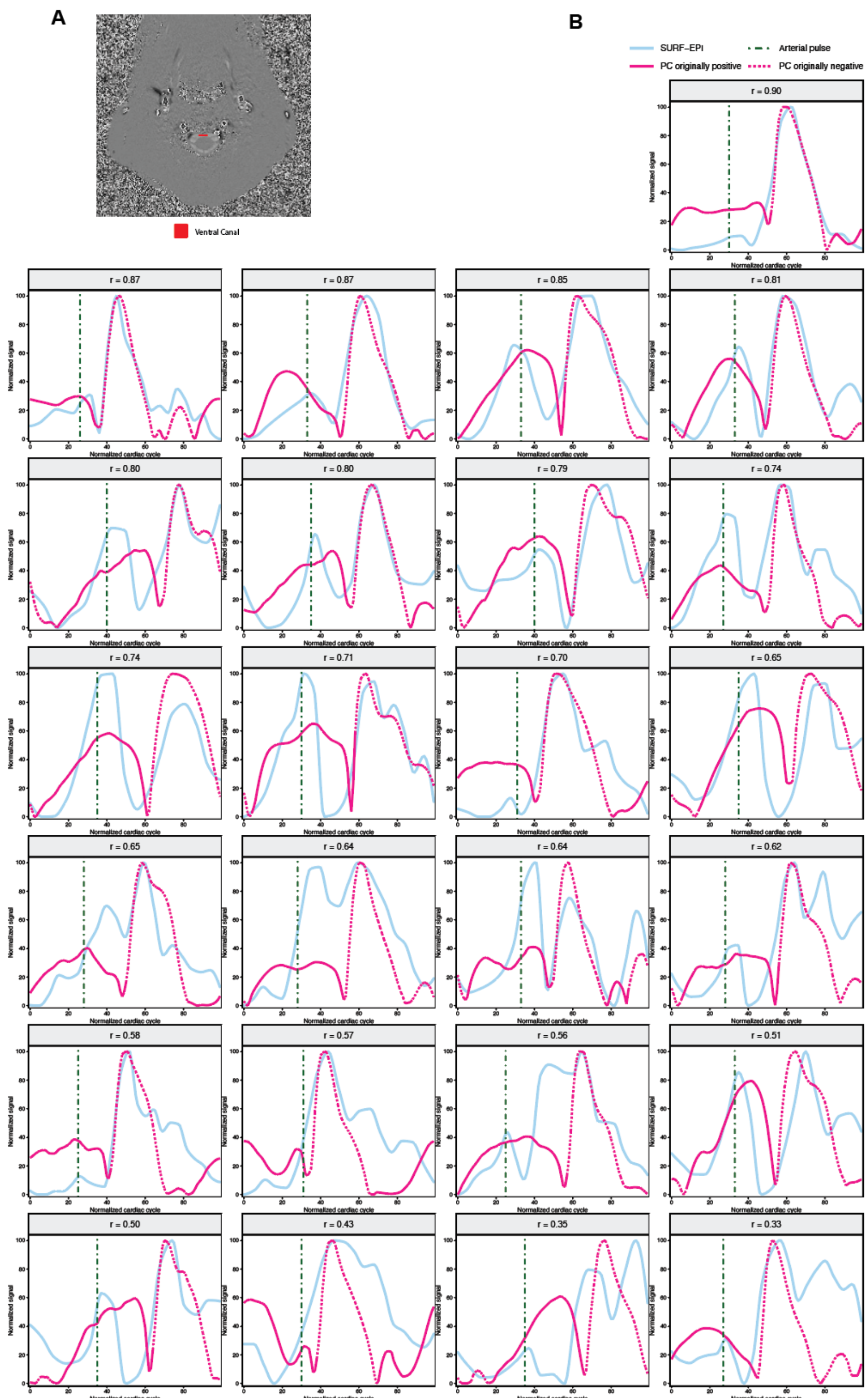

Figure S3. Participant-level agreement between ultrafast EPI and phase-contrast MRI CSF waveforms. (A) Phase-contrast MRI image acquired at C2–C3, with the ventral spinal canal region of interest indicated in red. **(B)** Cardiac-cycle CSF waveforms from all participants, ordered by the correlation coefficient between ultrafast EPI and phase-contrast MRI (PC-MRI). Ultrafast EPI signal is shown in light blue, and the originally positive and negative portions of the PC-MRI velocity waveform are shown by solid and dotted magenta lines, respectively. The arterial systolic peak used as a temporal reference is indicated by the green dash–dotted line. Waveforms were temporally aligned and normalized to their respective maxima; the participant-specific correlation coefficient is reported above each plot.

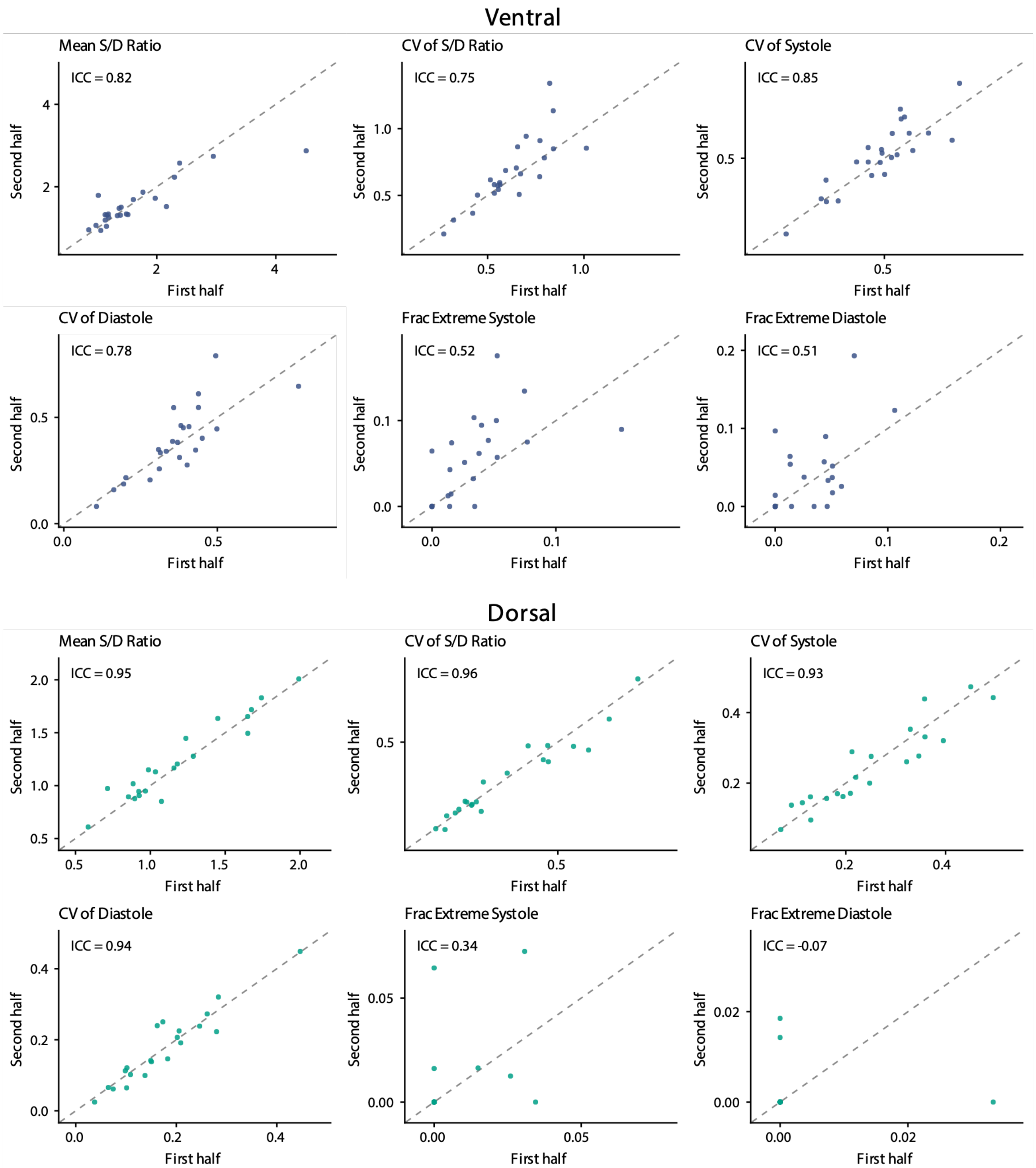

Figure S4. Split-half reliability of ultrafast EPI–derived cardiac-cycle metrics. Scatterplots compare measurements derived from the first and second halves of each acquisition for ventral CSF (*top*) and dorsal CSF (*bottom*). Metrics included the mean systole-to-diastole (S/D) ratio, coefficients of variation (CVs) of the S/D ratio, peak systolic signal and late-diastolic signal, and the fractions of cardiac cycles with extreme peak-systolic or late-diastolic signals. Each point represents one participant; dashed lines indicate identity. Split-half reliability was quantified using the intraclass correlation coefficient (ICC(2,1)), with metric- and compartment-specific ICC values shown in each panel.

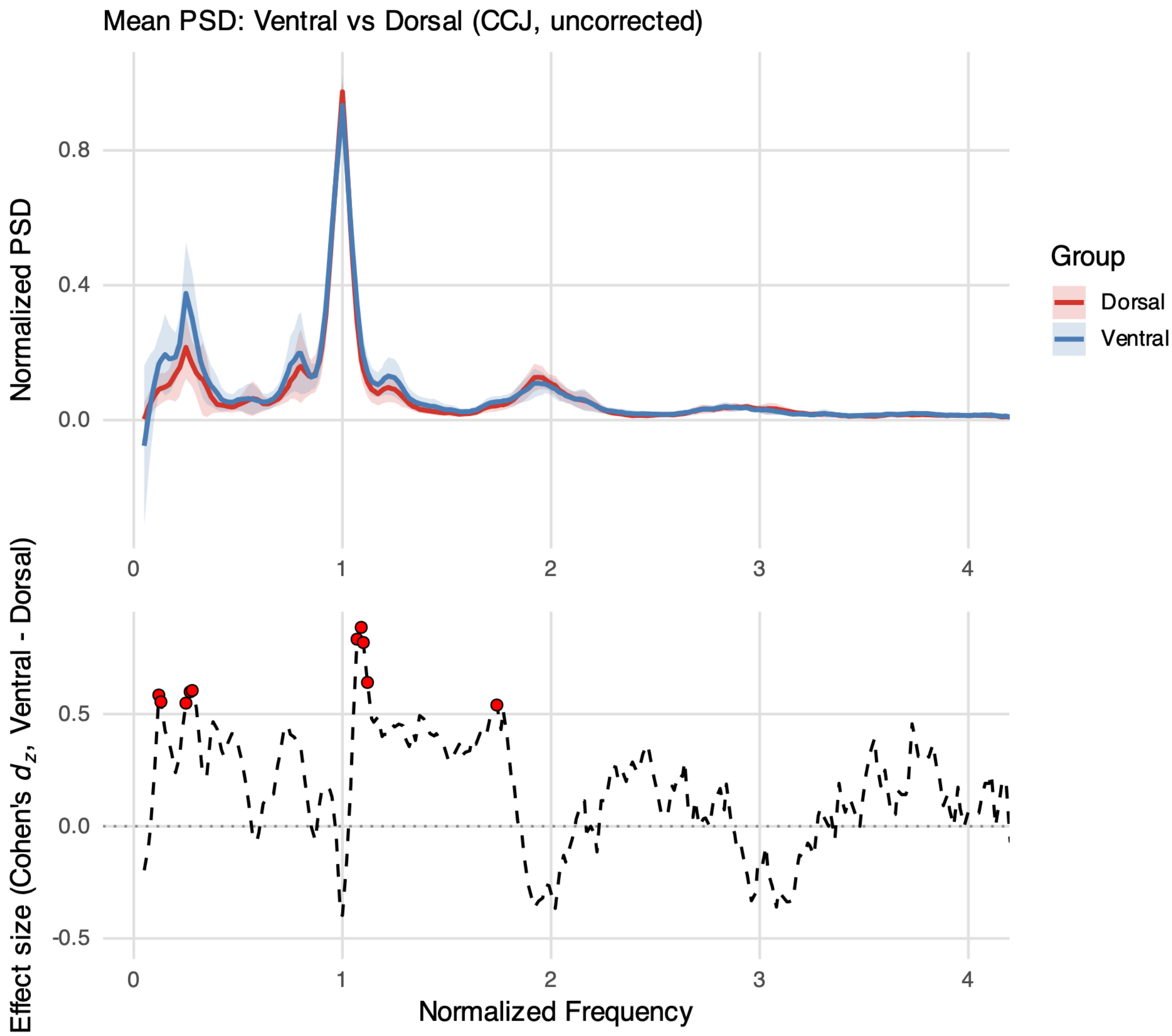

Figure S5. Frequency-dependent differences in ventral and dorsal CSF dynamics at the craniocervical junction. *Top*, group-averaged normalized power spectral density (PSD) of ultrafast EPI signals from ventral and dorsal CSF at the craniocervical junction (CCJ). *Bottom*, frequency-resolved Cohen's *d* effect sizes for ventral relative to dorsal CSF; positive values indicate greater power in ventral CSF. Colored markers denote nominal, uncorrected differences ($p < 0.05$); no frequencies survived false-discovery-rate correction. Frequencies were rescaled such that the cardiac fundamental frequency equaled 1 and the respiratory frequency was set to 0.25.

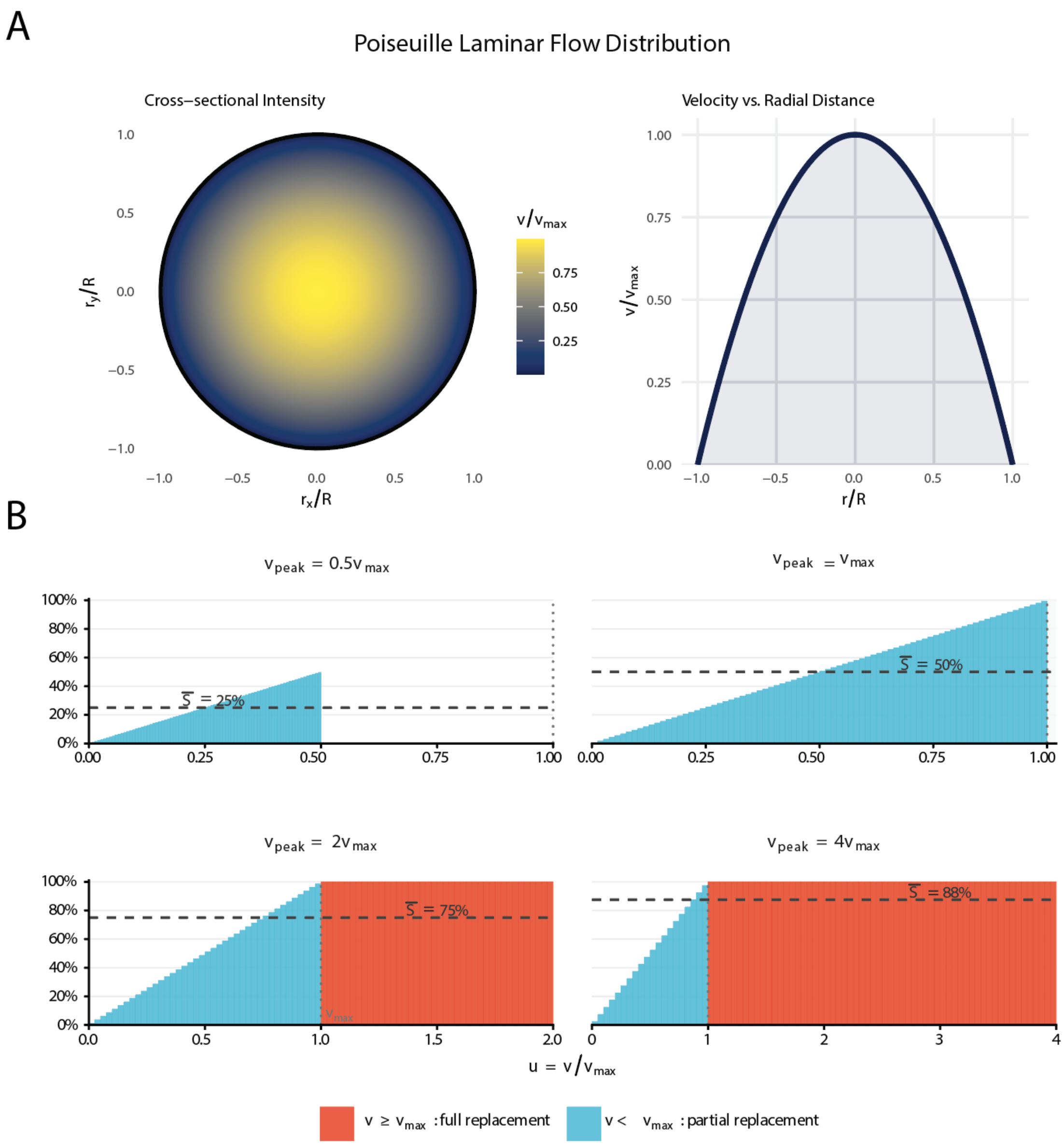

Supplementary Figure S6. Vascular inflow-weighted signal under Poiseuille laminar flow. **(A)** Cross-sectional velocity distribution and radial profile for laminar flow in a vessel oriented perpendicular to the imaging slice. **(B)** Predicted normalized inflow signal for $v_{peak} = 1/2 \cdot v_{max}$, v_{max}, $2 \cdot v_{max}$, and $4 \cdot v_{max}$, where $v_{max} = \Delta z/TR$ is the velocity required for complete spin replacement. Blue and red indicate partial and complete replacement, respectively.
